# Accurate identification of traps and pinch-outs on a stratigraphic reservoir-A case from Hala’alate Mountain in the Junggar Basin, China

**DOI:** 10.1371/journal.pone.0303467

**Published:** 2024-05-31

**Authors:** Xinshuai Li, Xuesong Yang, Huilai Wang, Chenlin Wu, Jun Xie, Qiongyao Pu, Xuecai Zhang, Xiaofan Hao, Yan Li

**Affiliations:** 1 College of Earth Science and Engineering, Shandong University of Science and Technology, Qingdao, China; 2 PetroChina Huabei Oilfield Bayan Exploration and Development Branch, Bayannaoer, China; 3 Shengli Oilfield, Dongying, China; Sapienza University of Rome: Universita degli Studi di Roma La Sapienza, ITALY

## Abstract

In the investigation of stratigraphic reservoirs, a significant discrepancy frequently exists between the delineation of the formation pinch-out line as traced using the characteristics of seismic wave reflections and the actual location of the formation pinch-out line. This has been the main problem restricting further hydrocarbon exploration and development. In this study, Hala’alate Mountain on the northwestern margin of the Junggar Basin is taken as an example for carrying out the study of stratigraphic reservoirs by integrating logging, drilling, and 3D seismic data. On the one hand, in studies based on the identification of formation pinch-out points using seismic data, the identification error of reservoir pinch-out lines is reduced by the improved included angle extrapolation method by utilizing the half energy attribute. On the other hand, the Poisson’s ratio curve is reconstructed using acoustic curves and oil-gas sensitive logging, then the reservoir oil-bearing facies zone is predicted using Poisson’s ratio post-stack genetic inversion to comprehensively analyze the controlling factors of stratigraphic reservoirs. The study area mainly features structural lithologic reservoirs, structural stratigraphic reservoirs and stratigraphic overlaps that pinch out reservoirs. The boundary of a stratigraphic reservoir is affected by the dip angle of the unconformity surface, the formation dip angle, and other factors. The improved included angle extrapolation method improves the identification accuracy of stratigraphic overlap pinch-out reservoirs. The reservoir distribution then is calculated according to Poisson’s ratio inversion, improving the prediction accuracy for the reservoir. This method improves the predictive effect for stratigraphic reservoirs and provides a new idea for the exploration and development of similar reservoirs.

## Introduction

Seismic interpretation technology, such as prestack migration imaging, seismic attribute analysis and reservoir prediction technology, is a key tool for complex hydrocarbon exploration in continental faulted basins and provides an effective means for predicting lithology and saturation heterogeneity, reservoir lithofacies thickness and reservoir attribute modeling [1–4]. The key to further improving the exploration accuracy for hydrocarbon reservoirs is to accurately identify the location of the formation pinch-out line. At present, using seismic data to study the scope of a formation pinch-out line generally involves extracting seismic attributes along the unconformity surface and then comparing and analyzing the results to determine the optimal attributes for identifying stratigraphic traps. For example, the instantaneous phase has unique advantages in tracking weak reflections with poor continuity and reflected waves with polarity changes and can be used to explain geological phenomena such as formation pinch out and horizon overlap [5]. In addition, geological statistics can be used to determine the thickness of strata in simulated nondenuded areas and extrapolated to determine the location of pinch-out points. Although seismic imaging technology has made great progress and is a common tool for detecting underground structures, seismic analysis of unconformity boundaries is still challenging due to the heterogeneity of rock physics. Therefore, the resolution of seismic data is extremely limited and this method can only roughly identify boundaries.

In basins around the world, researchers have adopted various methods to analyze hydrocarbon reservoirs such as petrophysical methods, facies analysis, and modeling [6, 7]. The study of rock physical properties is extremely important for determining reservoir mechanical properties. The plateau-derived porosity, permeability, and lateral connectivity of reservoirs also contribute to secondary hydrocarbon migration [8]. A fault zone increases the heterogeneity of petrophysics. The existence of weak hyperbolas in ungrinded seismic images is interpreted as evidence of a damage zone with low porosity relative to the host rock [9]. Moreover, basin analysis modeling is highly important for understanding hydrocarbon migration in fault systems [10–12]. The influence of regional tectonics on the sequence division of the study area is a prominent consideration in basin hydrocarbon exploration. The movement history and evolution model of regional tectonics have important impacts on the sedimentary model, horizon distribution and compaction evolution of basins [13–15]. In the Waldesar Basin, the interaction between regional tectonics and sea-level fluctuations plays a vital role in controlling accommodation space and sediment supply, and different correlations occur during the different stages of basin history. These analyses provide important scientific support for understanding oil reservoirs’ evolution process and resource distribution.

The northwestern margin of the Junggar Basin in Xinjiang is a complex structural area in the western uplift, formed by multistage tectonic movements. The structural characteristics of this area are mainly affected by the structural slope zone on the southern margin of Hala’alate Mountain. Due to these multistage tectonic movements, the strata in the study area unconformably overlie each other due to uplift and tilting. The Cretaceous strata unconformably overlie different strata of the underlying Jurassic (J), Triassic (T), Permian (P), and Carboniferous (C). In the Hala’alate Mountains, the high-angle unconformity is in the Carboniferous system and is pseudointegrated by the Tertiary system. Previous studies have extensively studied the southern margin of Hala’alate Mountain through basin analysis, sedimentary environment evolution, the relationship between structural characteristics and oil reservoirs, heavy mineral analysis and other methods [16–20]. Existing research on the relationships among hydrocarbon reservoirs has primarily explored the impact of structural changes on hydrocarbon accumulation, with a focus on the tectonic evolution of Paleogene and Cretaceous periods within continental sedimentary basins [16, 17, 21]. The formation of reservoir boundaries has always been an important bottleneck restricting hydrocarbon exploration. In this study, seismic profile and seismic attribute clustering are used for qualitative and semiquantitative research. Using well point statistics, the extrapolation error of the seismic data angle is controlled, and the position of the formation overbreak line is determined quantitatively. According to reservoir-forming factors such as structural morphology and sedimentary facies characteristics, the reservoirs on the southern margin of Hala’alate Mountain are dissected and analyzed, and the factors controlling the traps are discussed. This work provides a reference for the exploration and generation of other stratigraphic reservoirs worldwide.

## Geological overview

### Geologic set

**Fig 1:**
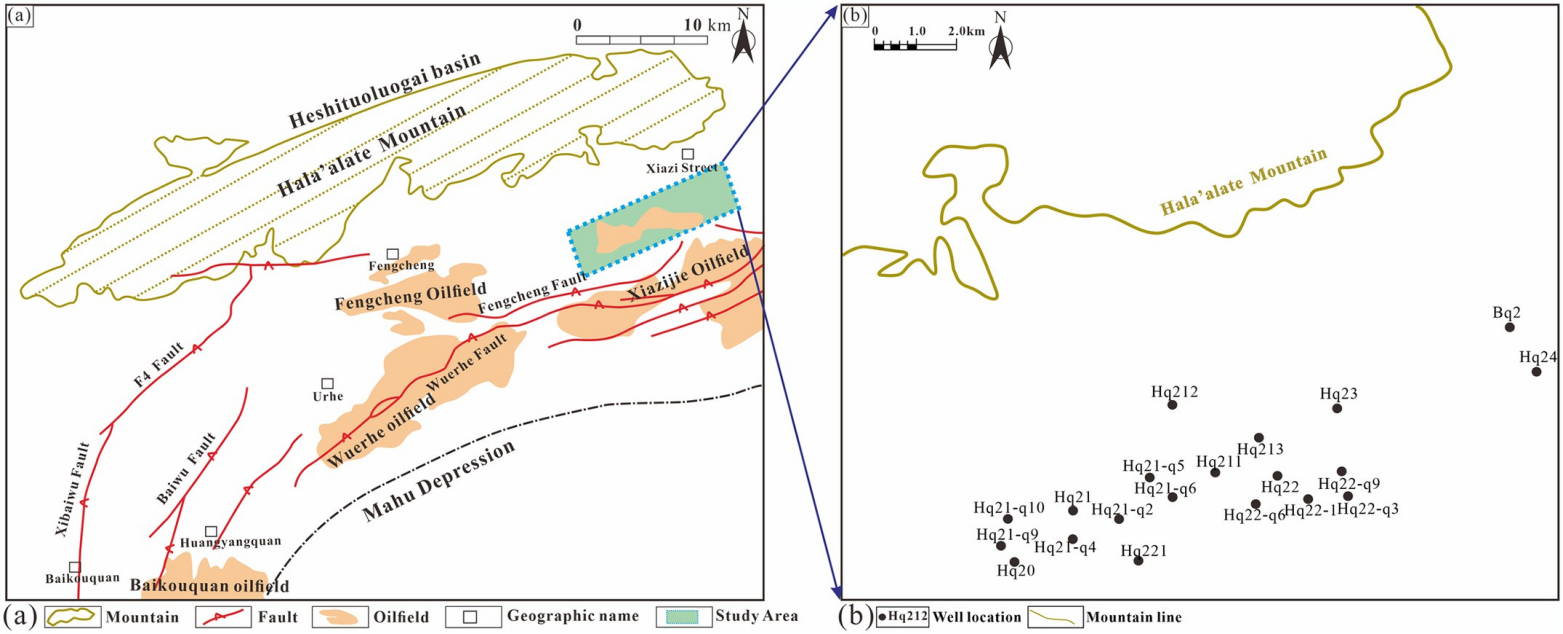
Hala’alate Mountain area on the northwestern margin of the Junggar Basin. (a) Hala’alate Mountain tectonic belt on the northern margin of the Junggar Basin. (b) Distribution map of well locations in the study area. Republished from ACS under a CC BY license, with permission, from Xiao Hu Dr. [24], the original copyright September 28, 2023.

The nappe thrust belt in the northwestern Junggar Basin is situated between the West Junggar fold mountains and the Junggar block. It is adjacent to the Mahu Sag to the south and bordered by Hala’alate Mountain to the north (Fig 1). During the Jurassic to Cretaceous, the Junggar Basin experienced filling and disappearance stages within an intracontinental depression. The lowermost Jurassic strata were influenced by the Triassic Indosinian movement, which resulted in the presence of widespread angular unconformities. Preserved formations in the middle and lower Jurassic include the Badaowan Formation, Sangonghe Formation, and Xishanyao Formation. However, the Toutunhe Formation and Qigu Formation from the Middle to Upper Jurassic are absent. The Jurassic system in the study area was impacted by the Yanshan movement, with only the Xishanyao Formation remaining. The Cretaceous and Jurassic strata are unconformable, with the development of the Qingshuihe [22, 23].

**Fig 2:**
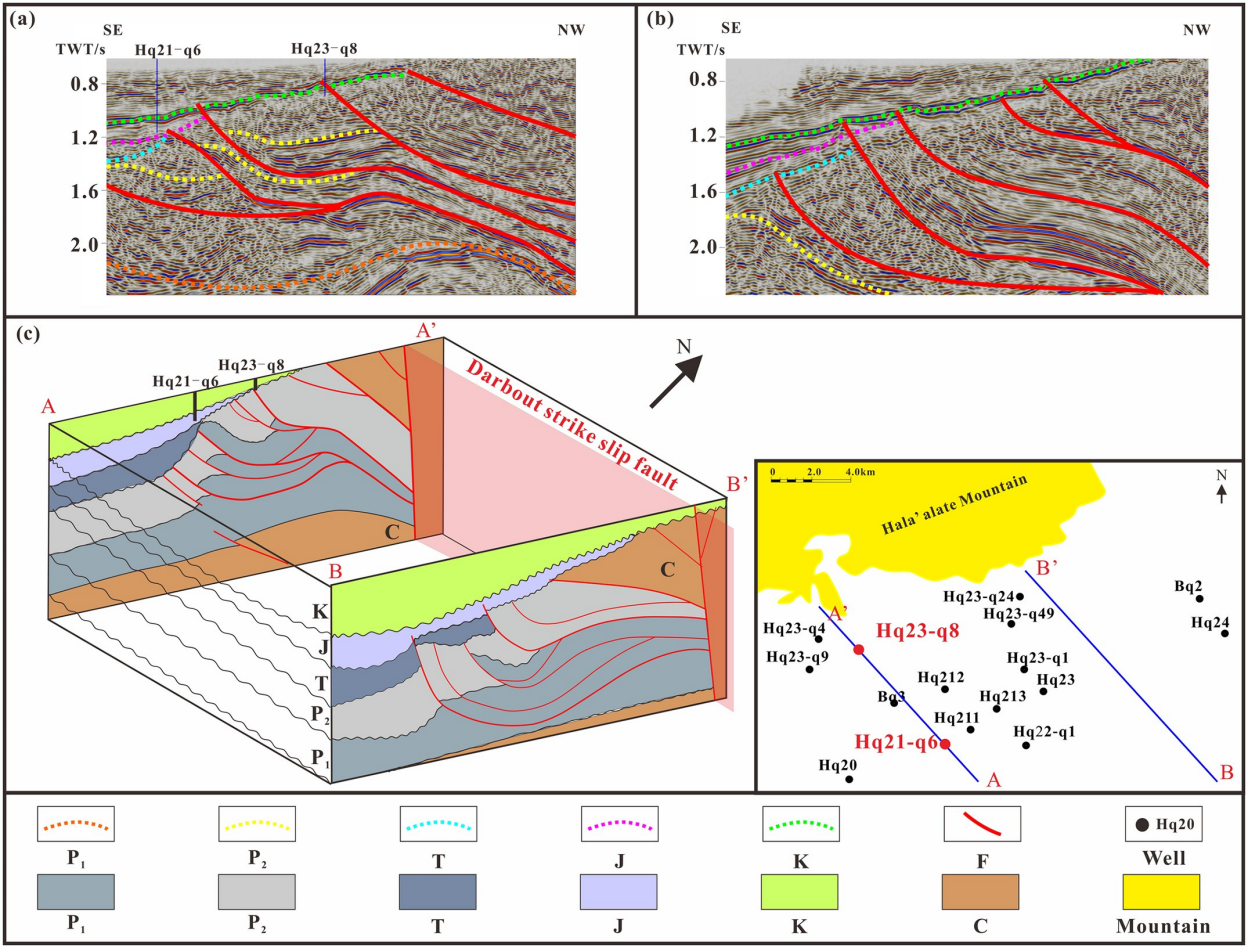
Comparison of east west structural characteristics in the study area. (a) The AA’ seismic profile from SE to NW shows the stratigraphic structure on the west side of the study area. (b) The BB’ seismic profile from SE to NW shows the stratigraphic structure on the east side of the study area. (c) Spatial display of the interpretation results for the AA’ and BB’ sections.

The Hala’alate Mountain Piedmont, situated on the northwestern margin of the Junggar Basin, is influenced by tectonic movement and exhibits paleotopography characterized by overlapping north-south structures and east-west uplift and depression. The deposits of the Xishanyao Formation display thickness variations from south to north, demonstrating east-west differentiation. The bottom boundary of the Jurassic system corresponds to the base of the first member of the Xishanyao Formation, while the top boundary is represented by the unconformable contact between the third member of the Xishanyao Formation and the Cretaceous lowermost unit (Fig 2). The Yanshanian movement underwent deceleration during the Cretaceous period. During this time, the uplift of mountains occurred alongside the cessation of faulting and folding activities. Consequently, the Cretaceous strata gradually overlapped in the vicinity of these mountains [25]. By comparing and analyzing seismic profiles AA’ and BB’ in the eastern and western regions of the study area (Fig 2a and 2b), the structural characteristics are characterized by upper and lower stratifications and east-west segmentation, respectively. The Jurassic strata are characterized by mainly composite waveform events with medium amplitude and poor continuity, and tectonic activity is relatively intense. In contrast, the Cretaceous strata exhibit obvious superelevation characteristics, moderate amplitude and good continuity. Compared with those in the east and west, the stratigraphic reflections in the east and west are relatively clear, and the stratigraphic contributions are clearer. Thrust faults are well developed in the west, and the superposition of multiple thrust faults leads to complex structural analysis. The eastern region shows weak tectonic activity, the dip angle of the fault is relatively small, and the vertical stacking is not intense (Fig 2c).

### Stratigraphy

**Fig 3:**
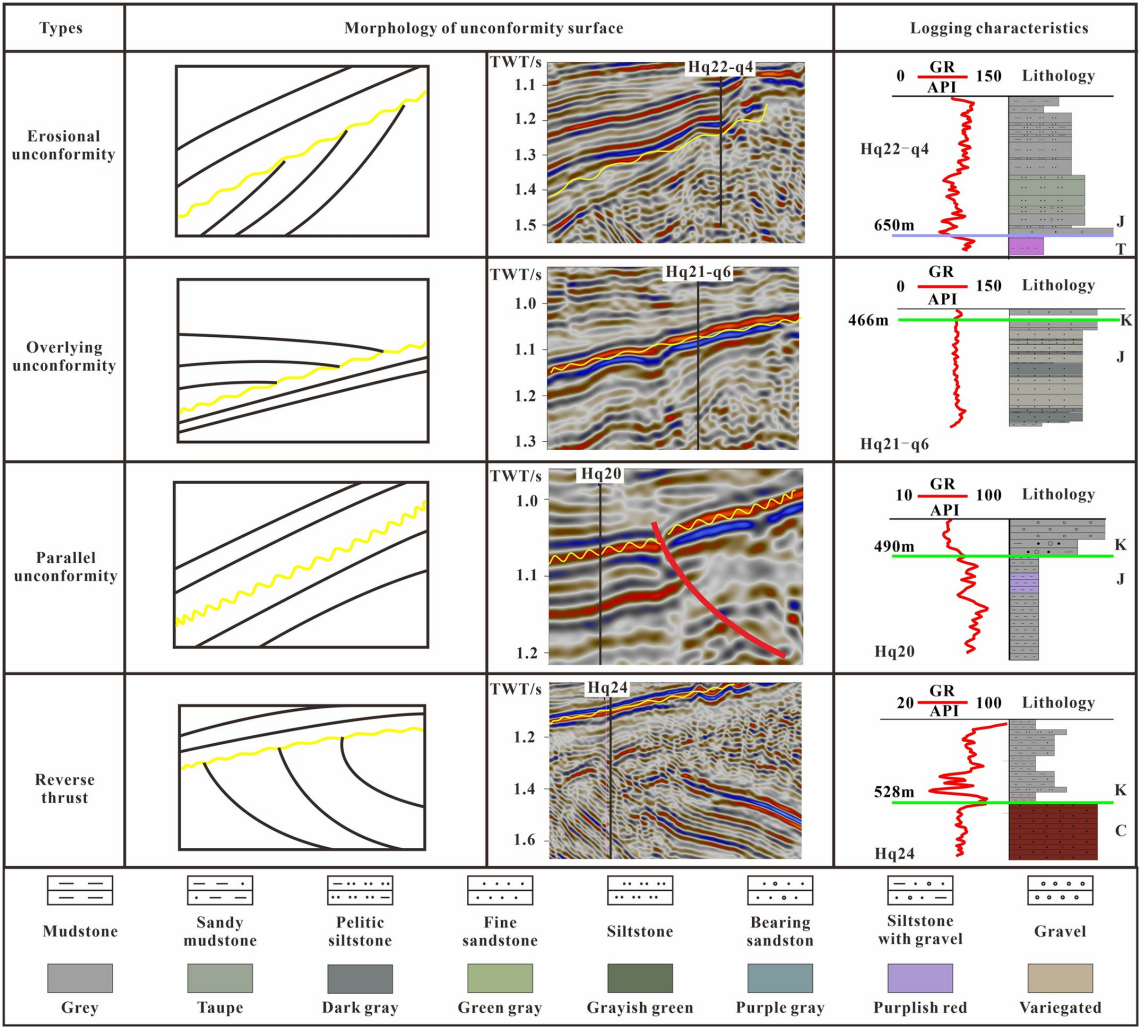
Stratigraphic contact relationships in the study area.

The distribution of stratigraphic reservoirs is controlled by unconformity surfaces, and the development characteristics of stratigraphic traps vary among different sequence interfaces and structural parts [26, 27]. By analyzing the termination characteristics of reflection event axes in three-dimensional seismic zones, various contact relationships can be identified. These relationships include erosional unconformity, overlying unconformity, parallel unconformity, and reverse thrust (Fig 3). In the southern region of the study area, a parallel unconformity is observed in areas characterized by thick strata and gentle slopes, which are located far from the provenance. The parallel unconformity surface between the Cretaceous and Jurassic systems primarily represents a lithological contact consisting of sand and mud, like Hq20. The Yanshan movement has resulted in an erosional unconformity between the Triassic and Jurassic systems, with a distinctive GR curve exhibiting a low and high funnel-shaped pattern, such as Hq21-q4; As we move further south, closer to the Piedmont slope zone and the provenance, the Cretaceous system demonstrates overlying unconformities, and the lithological variations between the upper and lower strata are minimal. The GR curve tends to resemble a baseline, as observed in Hq21-q6. In the proximity of the Piedmont, the Cretaceous strata persistently overlap with the Carboniferous, accompanied by the development of argillaceous intercalations within reverse thrust zones. The GR curve in this case exhibits a toothed or finger-shaped pattern, similar to Hq24 (Fig 3). The overlying traps were unrelated to the erosion unconformity and local erosion unconformity traps. Overlapping traps above uneven surfaces mostly developed around ancient and large uplifts, and the size of the reservoir facies was controlled by the sequence stratigraphy evolution of the overlying strata. Therefore, the identification of overlapping traps requires sequence stratigraphy research.

**Fig 4:**
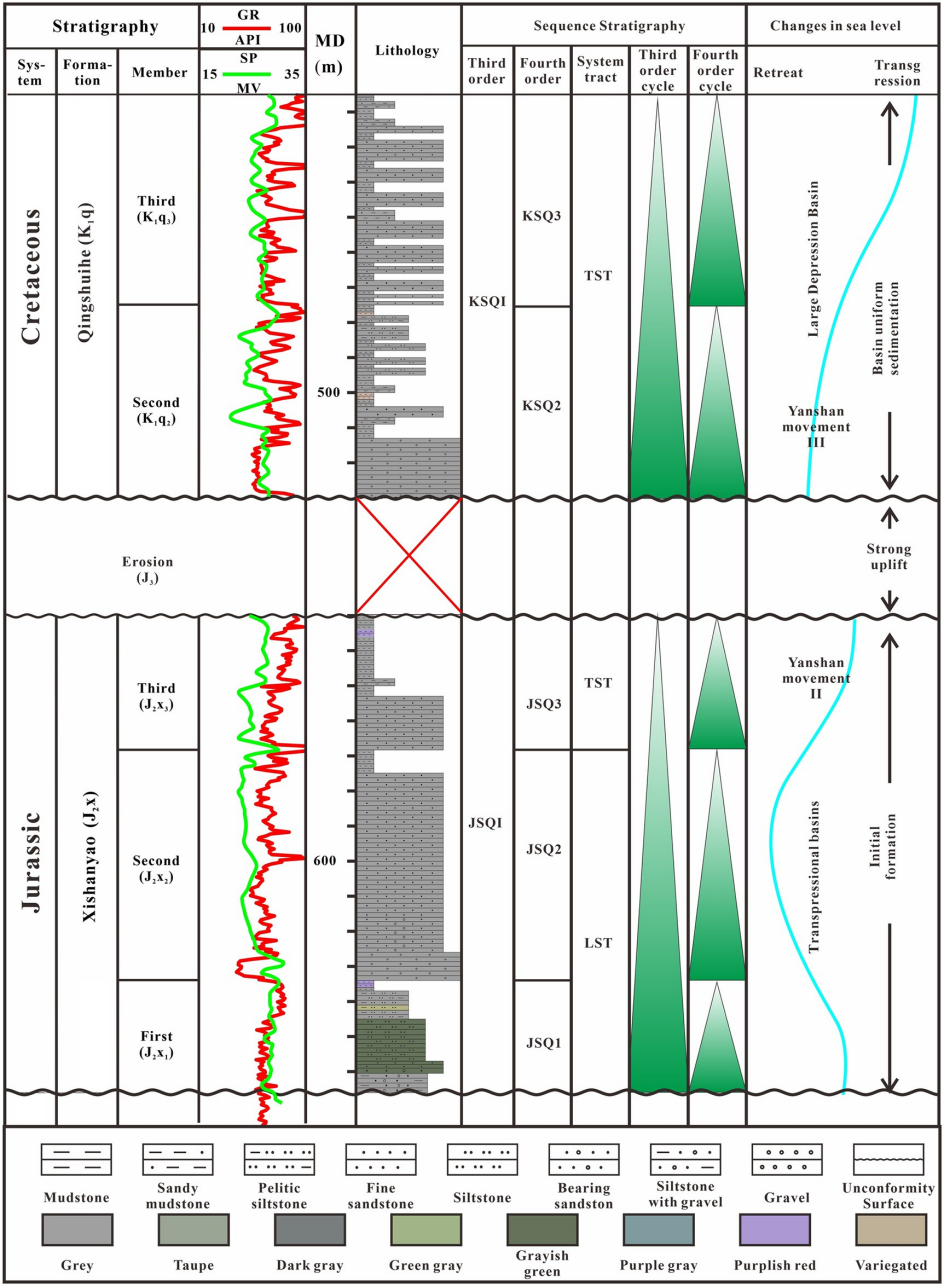
Comprehensive analysis diagram of sequence stratigraphy.

The previous division schemes of the Jurassic third-order sequence in the Hala’alate Mountain area are quite different [28–30]. The key to fine sequence stratigraphy research in this study is to identify the lake flooding surface and divide the system tract of the study area. Because the thickness of the fourth-order sequence within the third-order sequence in the study area is small, this sequence needs to be identified using lithology and logging. The Jurassic Xishanyao Formation is a third-order sequence (JSQI) [31, 32]. The seismic facies at the bottom of the Xishanyao Formation are easily identified by truncation. At the truncation point, the same phase axis is obviously merged, a truncation unconformity. The lithology of the first to second members of the Xishanyao Formation transitions from gray siltstone and fine sandstone to sandy conglomerate. In terms of electric logging, the natural gamma radiation is a box type, the natural potential is relatively flat, and the water depth decreases. The low stand tracts of the first and second members of the Xishanyao Formation are developed. A small-scale lake transgression occurred in the third member of the Xishanyao Formation. Sandy conglomerate occupied the lake bottom and developed a transgressive domain. Affected by the late Yanshanian movement, the basin uplifted and formed a high-level domain, with a large amount of erosion in the late stage and less residue in the high-level domain. The Cretaceous bottom boundary (KSQI) is characterized by the presence of a conglomerate layer, and the seismic facies in this area have strong amplitude, continuity, and parallelism. Each member of the Qingshuihe Formation exhibited a positive cycle from bottom to top, and the first member of the Qingshuihe Formation terminated in the basin. The lithology of the second member of the third member of the Qinghe Formation transitions from gray conglomerate to gray siltstone, and the natural potential and natural gamma field are of the dentate box type, indicating that the basin has experienced uniform subsidence and formed a relatively stable lacustrine transgressive system tract. In summary, the sequence division scheme of the study area was established. Two third-order sequences KSQI and JSQI were ultimately determined, as well as five fourth-order sequences KSQ2, KSQ3, JSQ1, JSQ2, JSQ3, corresponding to five members, respectively (Fig 4).

### Sedimentary characteristics

**Fig 5:**
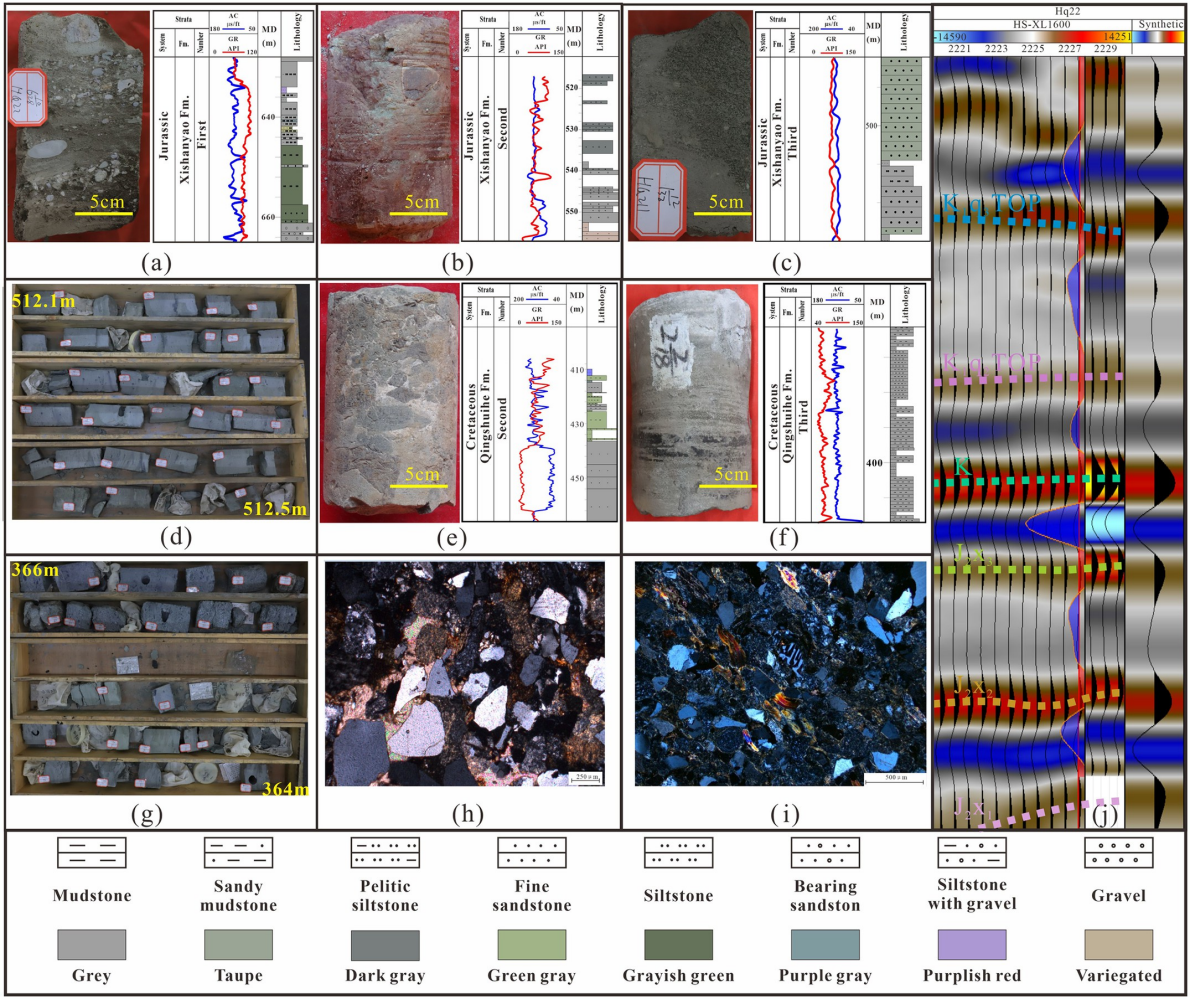
Characteristics of typical seismic profiles and typical core sequence boundaries in the study area. (a) Hq22, 661.36 m, pebbly argillaceous fine sandstone. (b) Hq21-q9, 554.70 m. Brownish red argillaceous conglomerate. (c) Hq211, 504.30 m, fine sandstone. (d) Hq211, 512.1~512.5 m, the mudstone of Xishanyao Formation is mainly gray. (e) Hq21-q6, 443.89 m, gray sandstone and conglomerate. (f) Hq23, 310.21 m gray siltstone. (g) Hq21, 364 m -365 m, The lithology of Qingshuihe formation is mainly grayish green mudstone and siltstone. (h) Hq20, 535.95 m, poor sorting and low roundness. (i) Hq21-q17, 467.91 m, poor sorting and low roundness. (j) Hq22, synthetic seismogram.

The sedimentary environment plays a decisive role in the process of hydrocarbon accumulation [33–36]. At the bottom of the Xishanyao Formation, there is thick grayish green and brown gravel, coarse to medium sandstone and gray to brown mudstone. The natural gamma ray is displayed as a toothed line shape, and the acoustic time difference is displayed as a small box shape (Fig 5a, 5b and 5d). In the third member of the Xishanyao Formation (JSQ3) a small-scale lake intrusion occurred resulting in an insufficient material supply and thinning of sandy gravel. The main interlayers are fine sandstone and siltstone with mudstone. The natural gamma curve and acoustic time difference plot as toothed lines (Fig 5c and 5d). There is obvious lithologies transformation between the Jurassic and Cretaceous strata. The lithology suddenly changed to a thick layer of bottom conglomerate, distributed throughout the whole area. The natural gamma curve and acoustic time difference exhibit a high-amplitude box shape, the upward lithology becomes finer, and sandstone interbedding is obvious. The natural gamma curve and acoustic time difference show a linear pattern (Fig 5e–5g). The Jurassic seismic facies exhibited a transition from low-amplitude, medium-amplitude, continuous reflections to nearly blank reflections, with strong-amplitude, continuous peak reflections occurring at the unconformity contact interface, and this gradually changed to nearly blank reflections in the Qingshuihe Formation (Fig 5j).

**Fig 6:**
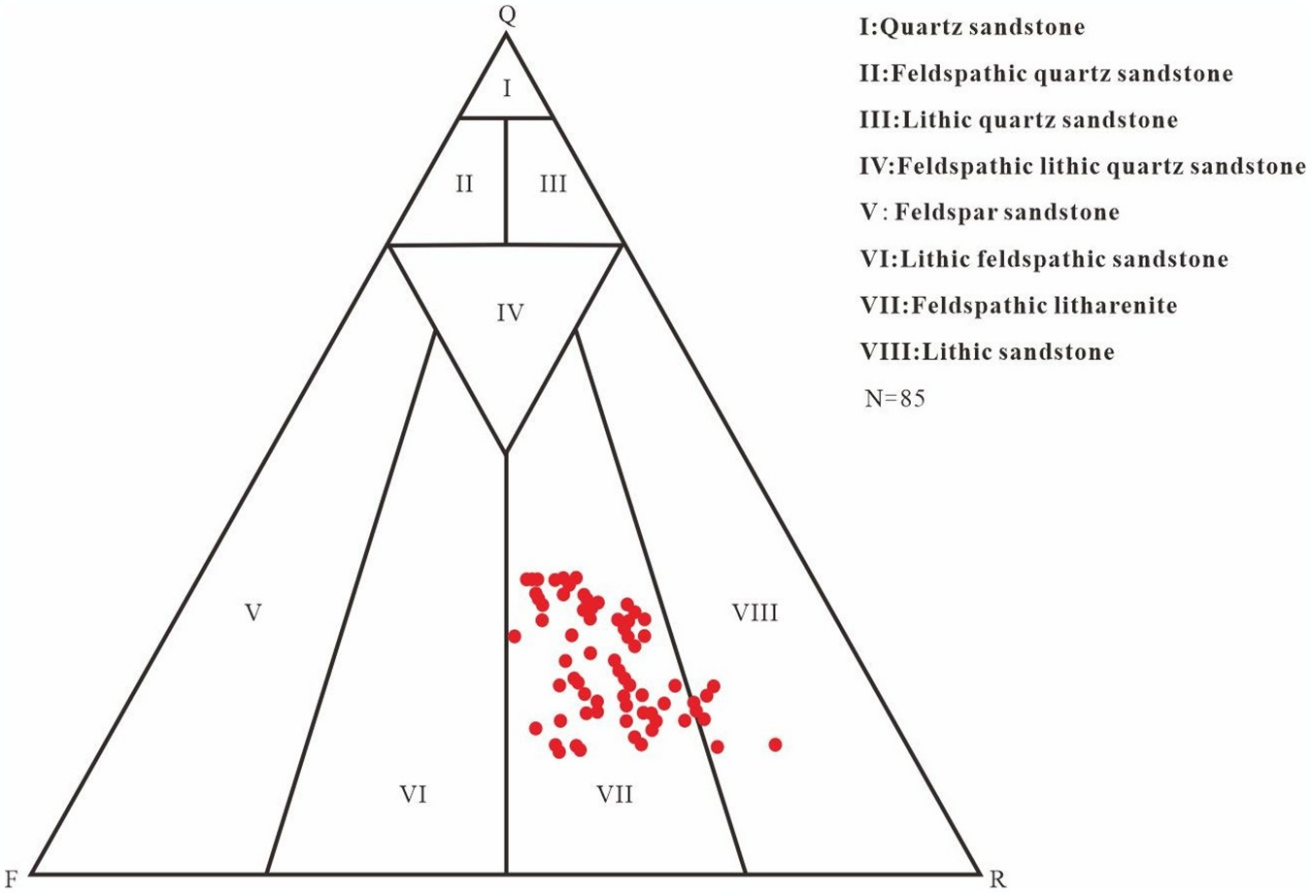
Triangle diagram of the Jurassic Cretaceous sandstone composition in the study area.

**Fig 7:**
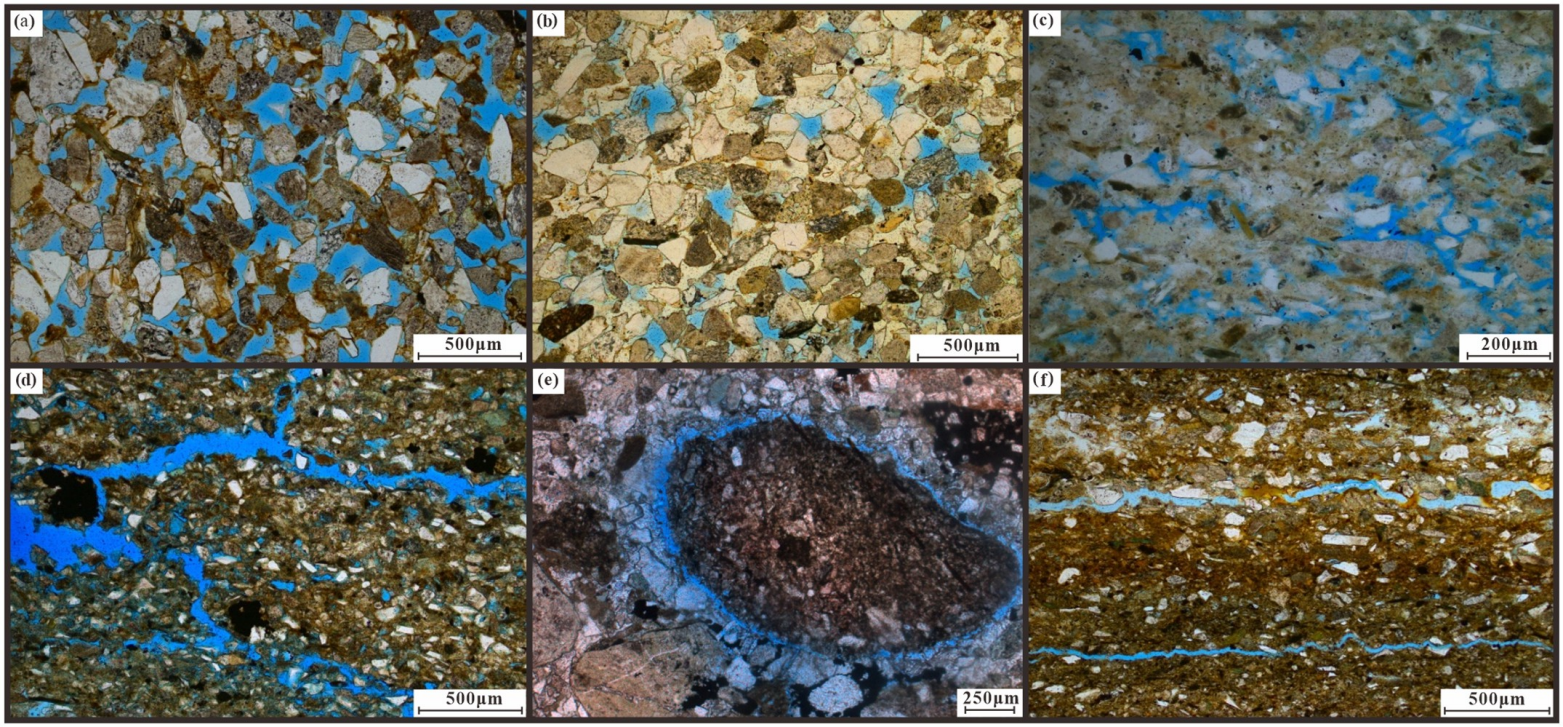
Type of storage space in the research area. (a) Hq23-q48, 135.27 m, primary intergranular pores. (b) Hq21-q10, 423.59 m, intergranular pores. (c) Hq23-q43, 87.05 m, intergranular pores. (d) Hq23-q43,87.05 m, intergranular dissolution crack. (e) Hq21, 443.76 m, with gravel joint. (f) Hq22-q10, 620.98 m, microcracks.

Identification of mineral composition and rock thin sections of 85 sandstone samples in the study area (Figs 6, 7 and Table 1), the content of feldspar particles ranges from 14.0% to 38.0%, with an average of 25.56%, and are mainly composed of alkaline feldspar and plagioclase feldspar. The range of quartz debris particles ranges from 15.0% to 35.0%, with an average of 25.59%. The distribution range of the rock debris content is 35.0% to 71.0%, with an average of 48.85%. The plastic rock debris content is high, and the particles are mainly in point line and line-line contact, with pore type calcite cementation as the main form. The analysis of the structure shows that the main types of sandstone in the study area are lithic sandstone and feldspar lithic sandstone rounded to subangular to subcircular shapes reflecting their low compositional maturity, good structural maturity, poor sandstone stability, short debris transport distance, rapid accumulation, and close sedimentary characteristics (Fig 7). The study area has developed fan delta sedimentation.

**Table 1:** Statistical table of rock mineral composition.

Mineral composition	Quartz (%)	Feldspar (%)	Rock debris (%)
Data statistics			
$\frac{Minimum-Maximum}{Average\;(sample\;number)}$	$\frac{15-35}{25.59(85)}$	$\frac{14-38}{25.56(85)}$	$\frac{35-71}{48.85(85)}$
Mode	30	23	47
Variance	44.96	21.49	66.39

## Methods

### Identification of the denudation boundary

The residual strata of the study area exhibit apparent unconformity contact relationships with second-order sequence boundaries. Notably, distinct variations can be observed between the attributes of strong wave groups and internal reflections. Thus, a comprehensive analysis of diverse seismic attributes effectively and accurately identifies and characterizes seismic horizons and delineates more precise boundaries for residual strata [37–39]. The 3D seismic data utilized in this study were collected at a sampling rate of 2 ms. The target interval exhibits a dominant frequency range of 20–25 Hz, with a bandwidth spanning from 10 Hz to 50 Hz, the resolution is around 26 m.

**Fig 8:**
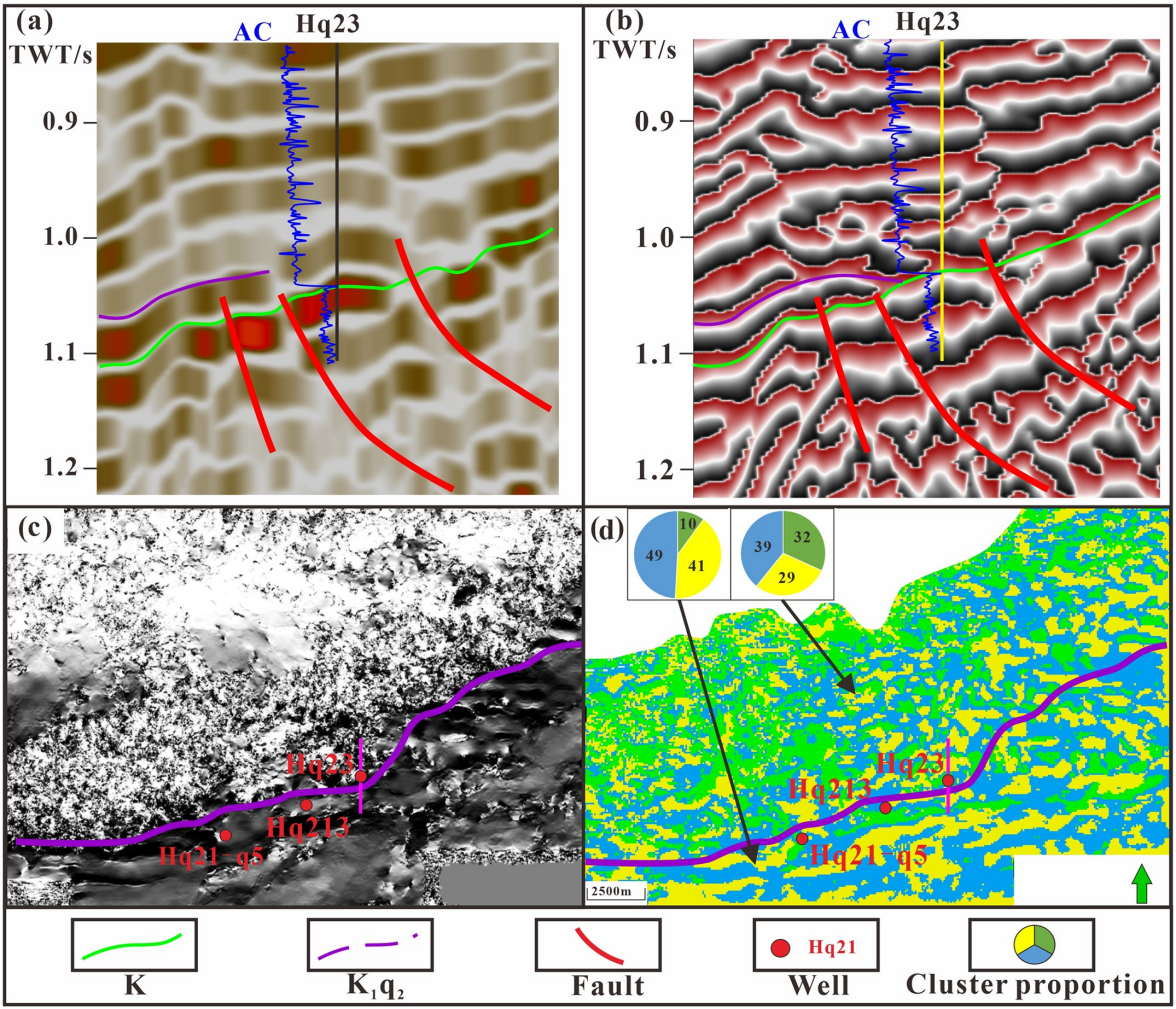
Multiattribute identification of stratigraphic boundaries. (a) Generalized spectrum decomposition. (b) Instantaneous phase. (c) Inclined body. (d) Attribute clustering.

Using a combination of various seismic attributes, formation dip angle changes, and seismic phase characteristics, the boundary is defined, enabling delineation of the overbreak point and distribution of horizontal residual strata [9, 38, 40–43]. General spectral decomposition (GSD) correlation calculations and instantaneous phase attributes prove to be particularly useful in determining the contact relationships between strata, providing a clearer representation of the structural relationships (Fig 8a and 8b). The Xishanyao Formation primarily exhibits truncation, whereas the Qingshuihe Formation demonstrates overlap, making it challenging to discern the reflection interface. The implementation of general spectral decomposition (GSD) correlation calculations can eliminate the negative correlation between the wavelet and seismic channel, resulting in an enhanced vertical resolution for a specific frequency. Additionally, the instantaneous phase attribute effectively represents the continuity energy strength of the event axis. Consequently, this approach proves valuable in distinguishing geological phenomena, including formation pinch-out, small faults, and horizon overlap. The identification of formation distribution boundaries can be achieved by assessing the discontinuity of the instantaneous phase or the presence of distinct boundaries in the phase plane in conjunction with drilling calibration. The dip angle attribute is derived from analyzing the dip time difference of each seismic channel, resulting in a sequence that highlights abrupt changes in dip angle, including faults, unconformities, and pinch outs. To supplement the reflection characteristics observed in isochronal slices with a dip angle of z = -1060 ms, drilling information is utilized. By examining multiple sets of slices, an approximate formation boundary is determined (Fig 8c). Seismic clustering is gradually becoming a new attribute analysis method. During the Cretaceous, this method can reflect changes in the sedimentary environment, lithology and lithofacies. The final seismic facies are divided into three categories: green, yellow, and blue (Fig 8d). The green type is mostly located in the northern region of the study area. Affected by the nappe of the Hala’alate Mountain front, most of the facies are seismic facies with poor continuity. The yellow and blue points are sandstone facies and mudstone facies deposited in a stable sedimentary environment, and most are parallel and subparallel seismic facies. According to the statistics of the proportion of seismic facies within and outside the sedimentary boundary of the second member of the Qingshuihe Formation, the proportions of yellow (41%) and blue (49%) seismic facies in the second member of the Qingshuihe Formation are relatively large, while the proportion of green (10%) facies is relatively small. The percentage of unstable green components (32%) during external deposition significantly increased, while the proportions of yellow (29%) and blue (39%) components decreased. A change in color reflects a change in the sedimentary environment, which provides a basis for us to determine the isochronous stratigraphic boundary.

**Fig 9:**
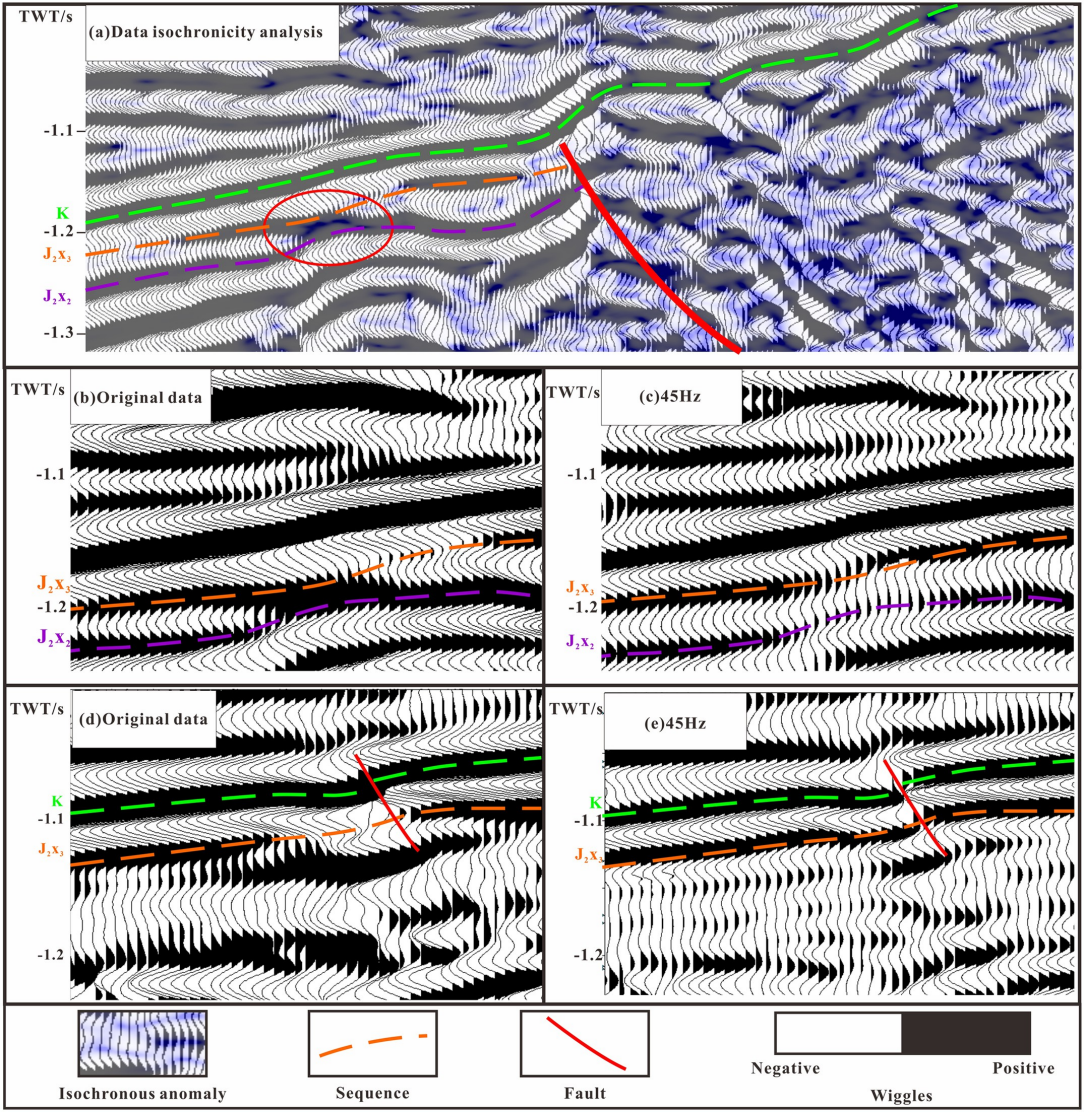
Layer tracing and structural interpretation of multi frequency seismic data. (a) Data isochronicity analysis. (b) Original data body and unequal reflection caused by changes in lithology. (c) 45 Hz data body, improving the unevenness caused by changes in lithology. (d) Original data body and distortion of the same phase axis near the fault zone. (e) 45 Hz data body and clear fault structure.

The accuracy of the description of the remaining strata is directly affected by the interpretation of the seismic layer data. In this study, seismic profile isochronicity analysis was performed on seismic data (Fig 9) to identify differences in wave propagation during seismic events. Then, multiscale wavelet decomposition technology was used to alleviate the unequal temporal characteristics of strata caused by waveform differences and the multiplicity of seismic interpretations. With the refinement of seismic research, the complexity of seismic reflections has gradually been recognized. In high-frequency data, seismic reflections tend to follow isochronous sedimentary interfaces, while in low-frequency data they tend to follow lithological interfaces [40]. Taking the north-south section of the research area as an example, areas with significant isochronous differences are shown in dark blue (Fig 9a), and the waveforms of the seismic event axes are mostly dominated by double-track erosion or lens-like motion. Two types of isochronous reflections were observed in the profile: reflections caused by lithological changes, which are not isochronous (Fig 9b), and reflections near faults (Fig 9d). When a reflection interface changes with seismic frequency, it is inaccurate [9, 44, 45]. Based on the above principles, by frequency division processing the amplitude intensity of the target main frequency range is highlighted while suppressing the amplitude response of other frequency, making the phase axis tracking more inclined toward the formation and more continuous (Fig 9c), making the structural information more detailed and the faults clearer (Fig 9e).

### Improvement of the angle extrapolation method based on energy attributes

**Fig 10:**
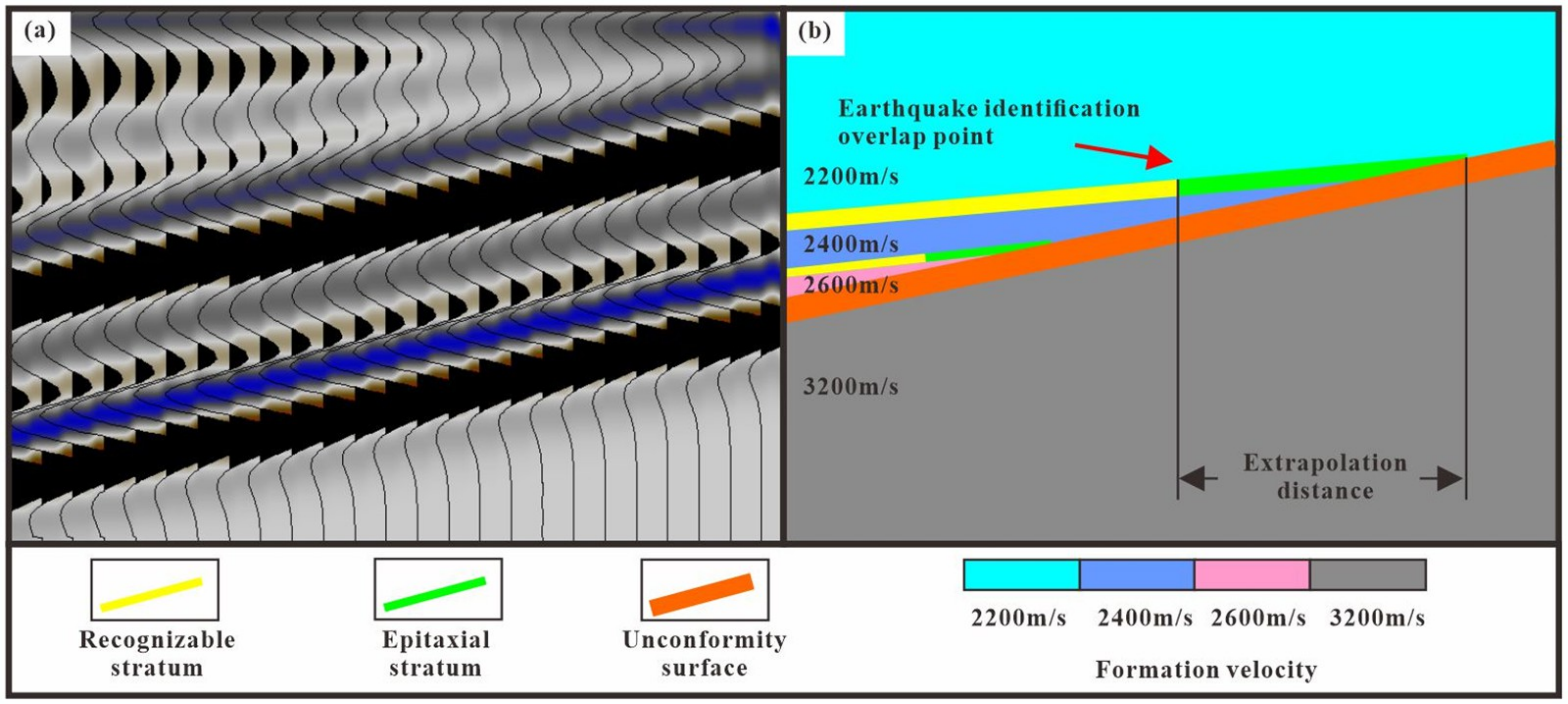
Seismic forward profile (a) and stratigraphic overlap Model (b).

Based on meticulous seismic interpretation, a multitude of seismic attributes are employed to identify the planar distribution characteristics of the Cretaceous overlap line and the Jurassic truncated line. This approach enables the determination of the approximate extent of unconformities and lithologic traps within a single evaluation. Nonetheless, seismic data impose limitations, resulting in a certain degree of separation between the eroded and overlapping boundaries as recognized by seismic attributes and actual boundaries. Therefore, a seismic tectonic model of the study area is established and the seismic response characteristics of the unconformity are verified by two-dimensional seismic forward modeling [46–49]. The forward modeling parameters are selected as follows: sandstone velocity, 2400 m/s; mudstone velocity, 3200 m/s; and seismic dominant frequency, 25 Hz (Fig 10a). The identification of pinch points on seismic profiles relies mainly on the amplitude characteristics of the event axis. When the thickness of the sand body in the formation model is greater than one fourth of the wavelength, the thickness of the sand body is greater than 24 m and the wave crest and trough are completely separated. With the formation overlapping the formation thickness is reduced to one fourth of the wavelength and the formation thickness in the model is less than 24 m. The sand body cannot form an independent reflected wave and cannot be separated from the reflection of the unconformity (Fig 10b). Therefore, in theory the reflection termination point of seismic data identification is mostly near the quarter wavelength. In practice, this approach is often affected by changes in seismic data and geological conditions so it is impossible to accurately determine the position of the actual overlap line.

**Fig 11:**
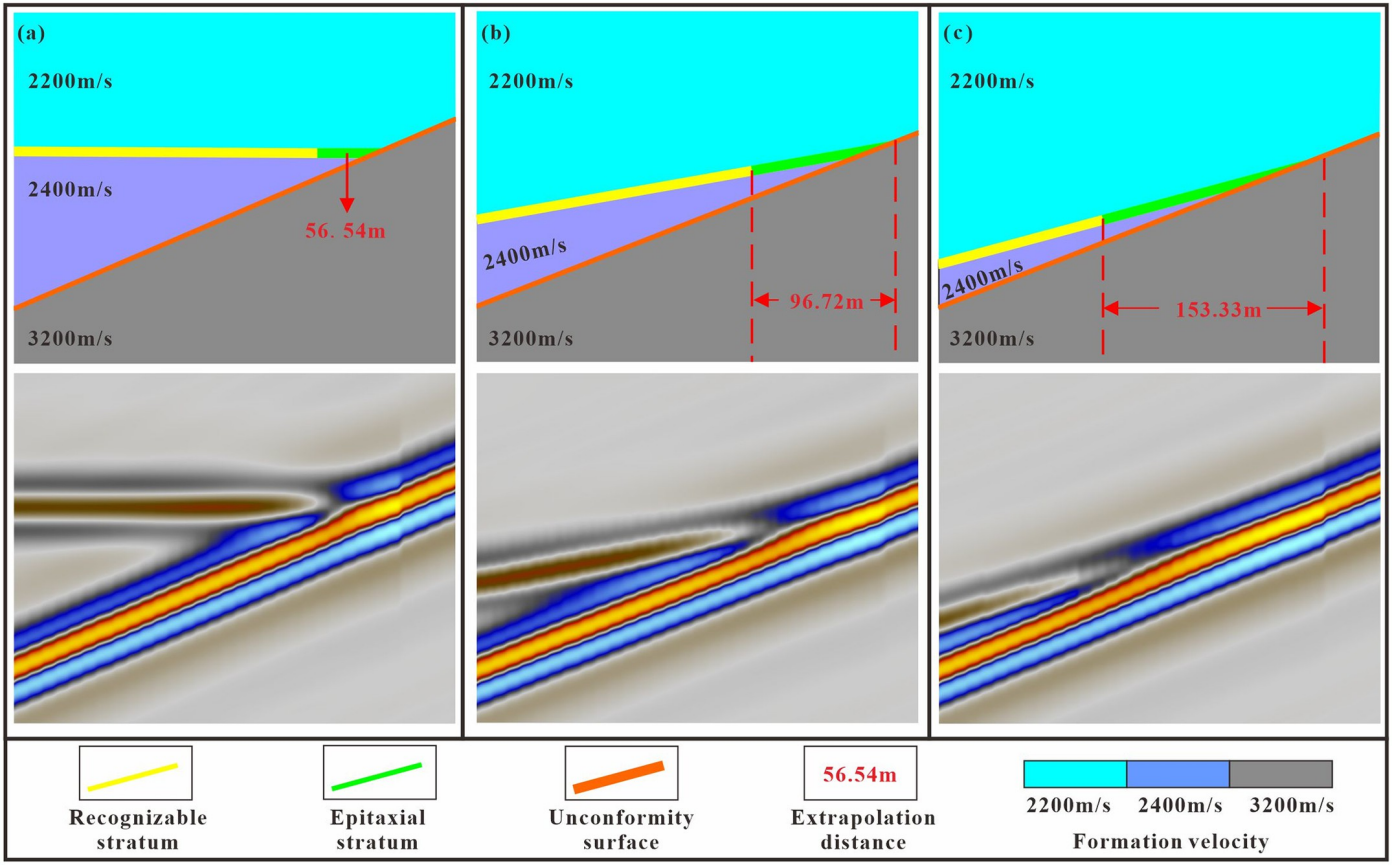
Stratigraphic overlap model (top) and seismic forward profile (bottom).

The distance between the identified points of overlap and denudation and the actual points is influenced by various factors, including the formation’s dip angle, velocity, and the dominant frequency of the seismic wave. A decrease in layer velocity correlates with a reduction in extrapolation distance. Similarly, an increase in the formation dip angle leads to a shorter extrapolation distance (Fig 11). Therefore, in this study an extrapolation empirical formula is used to precisely determine the positions of the stratigraphic overlap and denudation points (1). Since the extrapolation distance cannot be directly observed in seismic data, the wavelength is calculated based on the propagation velocity and seismic frequency. The limiting resolution of the seismic data is then determined to be one-fourth of the wavelength. By combining this information with the layer’s dip angle the extrapolation distance can be obtained. Petrel software was used to calculate the dip angle of the layers.

Unconformity dip of 23°. (a) Formation dip of 0°. (b) Formation dip of 10°. (c) Formation dip of 15°.

$$x=\frac{\text{V}\;\text{cos}\left(a_{1}\right)\text{cos}\left(a_{2}\right)}{4\text{f}\;\text{sin}\left(a_{1}-a_{2}\right)}$$ (1)

where x—extrapolation distance, m; f-dominant frequency, Hz; a_1_- dip angle of unconformity surface, °; a_2_- overburden dip angle, °; V—formation velocity, m/s.

**Fig 12:**
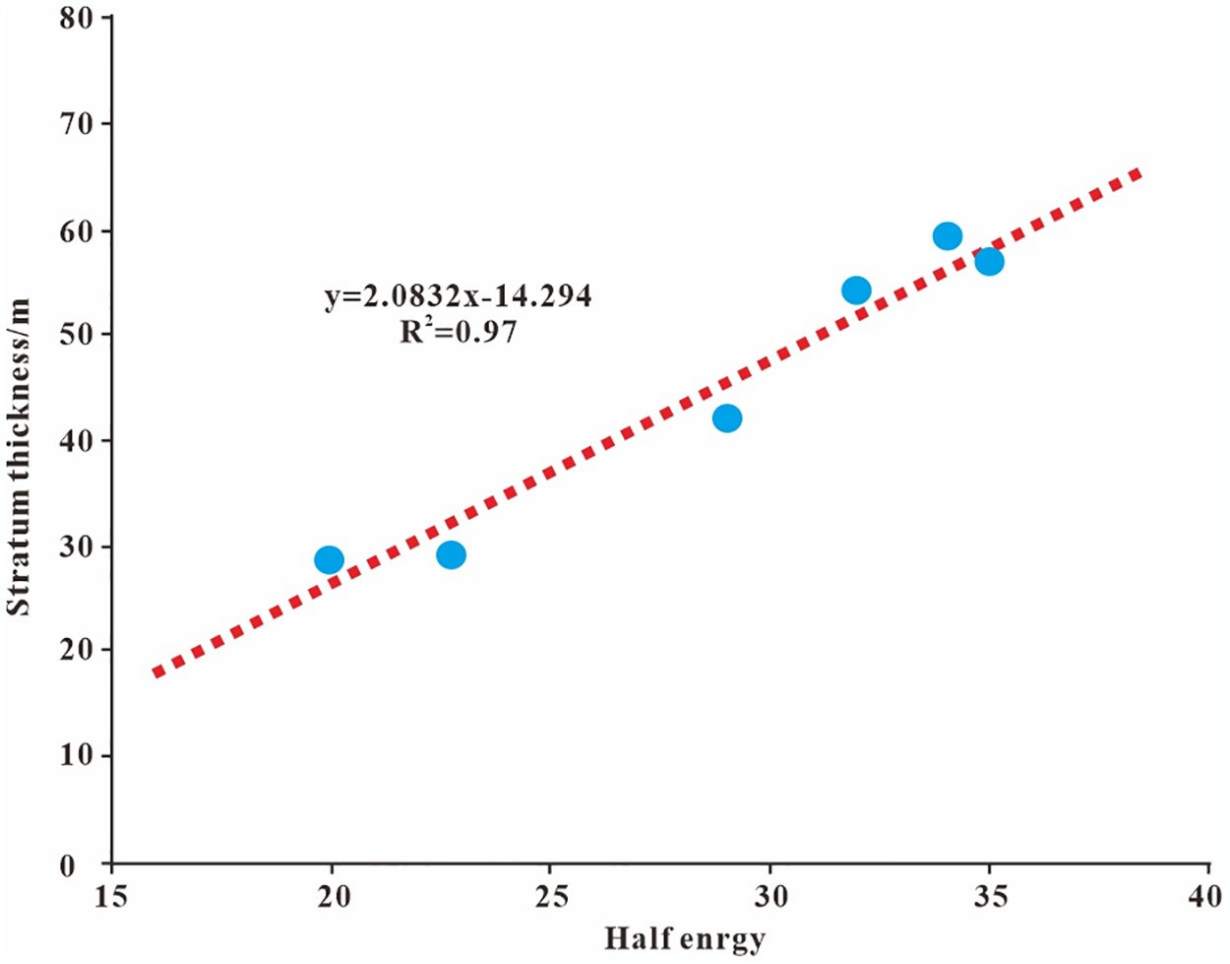
Relationship between drilling thickness and half energy in the second member of the Qingshuihe Formation.

The study area overlaps with the Piedmont, and the residual formation thickness is thin and variable greatly. According to the drilling data, the thickness of the second member of the Qingshuihe Formation is within this stratum and the lithology is highly heterogeneous. Therefore, seismic attributes are used to perform correlation fitting (Fig 12) to predict the residual formation thickness. The thickness correction coefficient C (2) is obtained by comparing the formation thickness predicted by the fitting formula with the drilling formation thickness to improve the horizontal heterogeneity of the formation and lay the foundation for subsequent formation reservoir prediction.

$$C=\left(\frac{H_{P}}{H_{T}}\right)-1$$ (2)

where H_T_ is the drilling thickness, m; H_P_ is the predicted thickness, m; and the C is the correction coefficient, dimensionless.

When the predicted value coincides with the true thickness no correction is needed and the coefficient C is 0. If the predicted value exceeds the true thickness C is a positive number. If the predicted value is smaller than the true thickness, C is negative. The true thickness can be determined using logging data or utilizing the time-depth relationship of well seismic combinations (Table 2). The predicted thickness is obtained through the fitting relationship between half energy and formation thickness (Fig 12). To improve the results of the formula the measured values are refined by considering the limiting resolution of the seismic data (3).


$$x=\frac{\text{v}\;\text{cos}\left(a_{1}\right)\text{cos}\left(a_{2}\right)}{4\text{f}\;\text{sin}\left(a_{1}-a_{2}\right)\left(1+c\right)}$$ (3)

**Table 2:** Table of statistics for predicting the thickness of the second memner of the Qingshuihe Formation.

Well	Thickness/m		C
	H_T_	H_P_	
Hq22	54.5	52.3684	-0.039
Hq24	42	44.0356	0.048
Hq21-q6	59.5	56.5348	-0.050
Hq22-q7	28	31.5364	0.126
Hq22-q10	57	58.618	0.028

### Poisson’s ratio genetic inversion

In seismology, the ratio of the longitudinal wave velocity of the transverse wave velocity can be used to determine Poisson’s ratio. In hydrocarbon seismic exploration, the prestack seismic inversion method is usually used to obtain Poisson’s ratio [50–53]. Given the longitudinal wave velocity Vp and transverse wave velocity Vs, Poisson’s ratio can be obtained through the intrinsic relationship of the elastic parameters (4):

$$\nu =\frac{v_{p}^{2}+2v_{s}^{2}}{2v_{p}^{2}-2v_{s}^{2}}$$ (4)

where ν—Poisson’s ratio, dimensionless; Vp—P-wave velocity, m/s; Vs—S-wave velocity, m/s.

Generally, a low range of Vp/Vs is more likely to correspond to better reservoirs. Within this range, Poisson is more sensitive and has a greater degree of differentiation. Vp/Vs can usually be obtained using the prestack seismic inversion diameter, but geologists often cannot obtain prestack seismic data according to the diameter. Therefore, the longitudinal wave velocity Vp and transverse wave velocity Vs can be calculated based on the characteristics of the acoustic time difference data (5):

$$v_{p}=\frac{3.048\times 10^{5}}{\text{Dt}}$$ (5)

where Dt-interval transit time, s/m.

When calculating the shear wave velocity, it is necessary to consider the influence of different rock cores on the velocity propagation. When there is rock debris logging data, the lithology can be directly distinguished using the Greenberg–Castagna (GC) formula [54]. When the lithology is unknown, the GR curve can be used for lithology classification. When the GR content is less than 80 the facies tend to constitute sandstone, and when the GR content is greater than 80 the facies tend to constitute mudstone (6) and (7).


$$\text{Sandstone}:\;v_{s}=0.8416*v_{p}-0.8588$$ (6)

$$\text{Mudstone}:\;v_{s}=0.7697*v_{p}-0.8674$$ (7)

**Fig 13:**
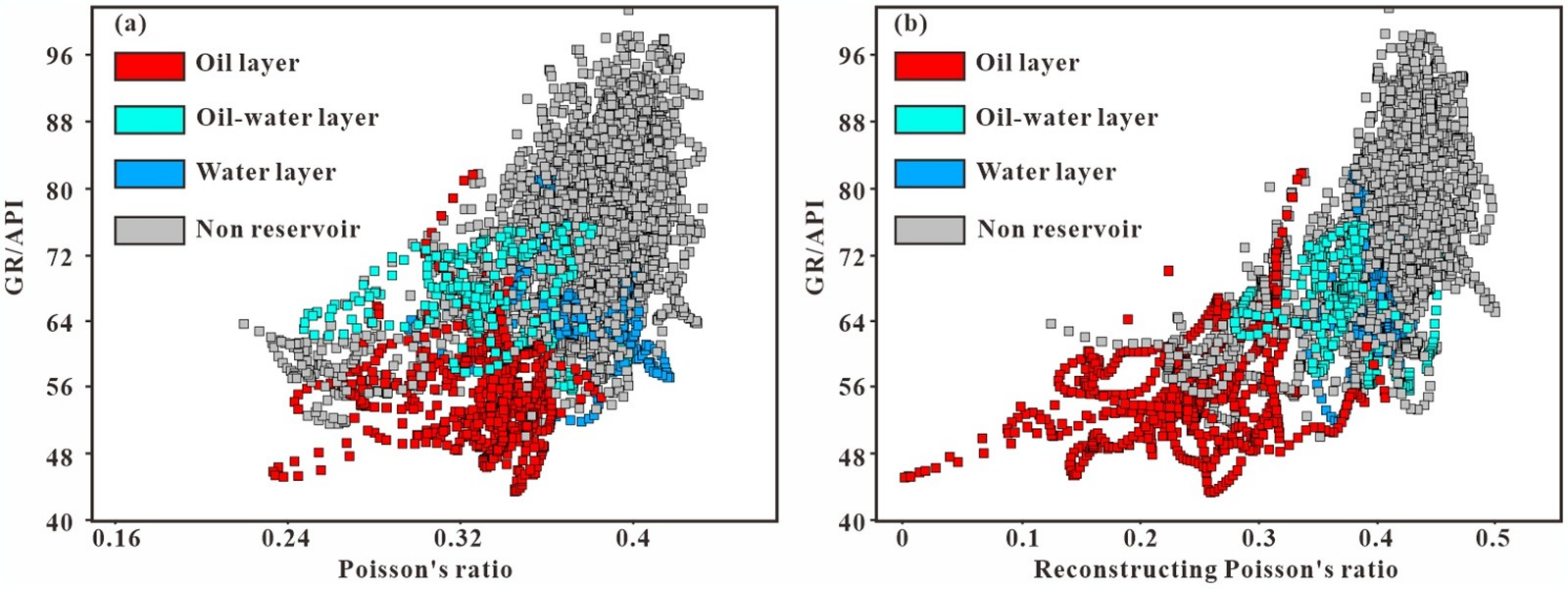
Poisson’s ratio GR electric logging intersection diagram.

Poisson’s ratio can be used to distinguish between sandstone reservoirs and mudstone cap rocks effectively. According to the intersection diagram, a threshold value for the reservoir can be set, which is advantageous for reservoirs: a Poisson’s ratio of 0.0~0.3 (Fig 13a). The analysis results were compared with the well data within the work area and it was found that the oil layer and dry layer have good differences in terms of Poisson’s ratio values, but the water layer and oil layer are difficult to distinguish. Therefore, in this Poisson’s ratio inversion the multicurve pseudoacoustic method was used to reconstruct the Poisson’s ratio curve, normalize GR, CAL, and R25, and multiply them by weight coefficients of 0.3, 0.25, and 0.6, respectively (8) and (9). Then, Poisson’s ratio was used to add or subtract based on the positive or negative correlation with the curve, and finally, the Poisson’s ratio was normalized to complete the reconstruction (10). After reconstruction, the distribution of favorable reservoirs can be visualized (Fig 13b).

(a) Intersection diagram of Poisson’s ratio and GR. (b) Reconstruction of the intersection graph of Poisson’s ratio and GR.

$$A_{N,i}=\frac{A_{i}-A_{min}}{A-A_{max}}$$ (8)

$$K_{i}=w_{i}*\left(A_{N,i}-\bar{A_{N,\;i}}\right)$$ (9)

$$R_{i}=(1\pm K_{i})$$ (10)

where *A*_*N*,*i*_—Value of each sampling point of the normalized logging curve, dimensionless; *A*_*i*_—Logging curve sampling point value, dimensionless; *Amin*, *Amax*-Minimum and maximum values in sample points, dimensionless; $\bar{A_{N,i}}—$ Normalized average, dimensionless; *Wi*—weight coefficients, dimensionless; *K*_*i*_—Weighted quantity, dimensionless; *R*_*i*_—Reconstruction curve, dimensionless.

**Fig 14:**
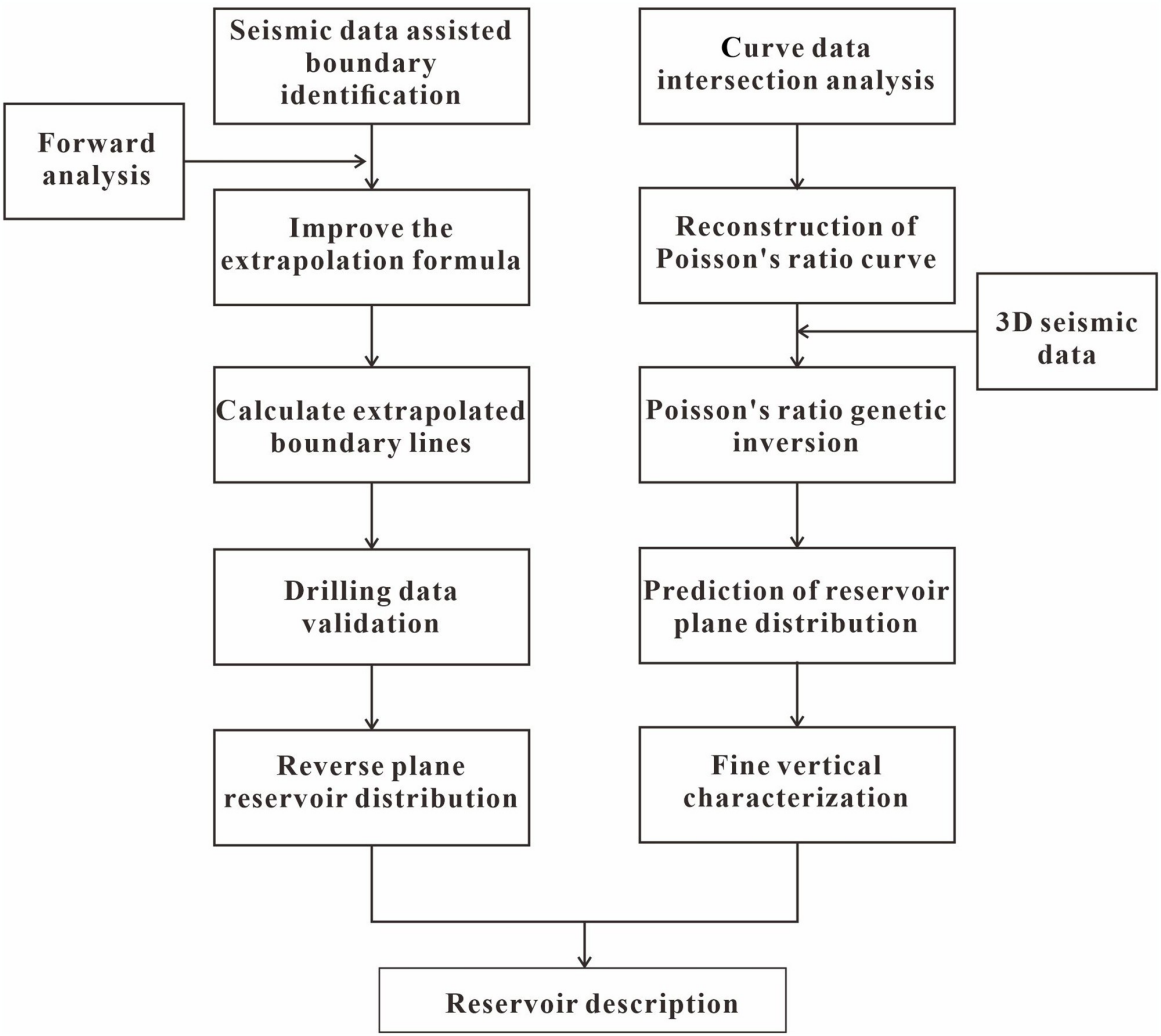
Flowchart of the Poisson’s ratio inversion technique.

When prestack seismic data cannot be obtained, the nonlinear relationship between the Poisson’s ratio curve and seismic data can be explored via a BP neural network. Unlike AVO inversion, genetic inversion does not require an *a priori* model or wavelet as input but rather uses a multilayer neural network + genetic algorithm to complete the inversion process. In summary, we completed the study of favorable blocks in the target area by extrapolation of the stratum angle and genetic inversion of Poisson’s ratio (Fig 14).

## Results

### Main controlling factors of traps

**Fig 15:**
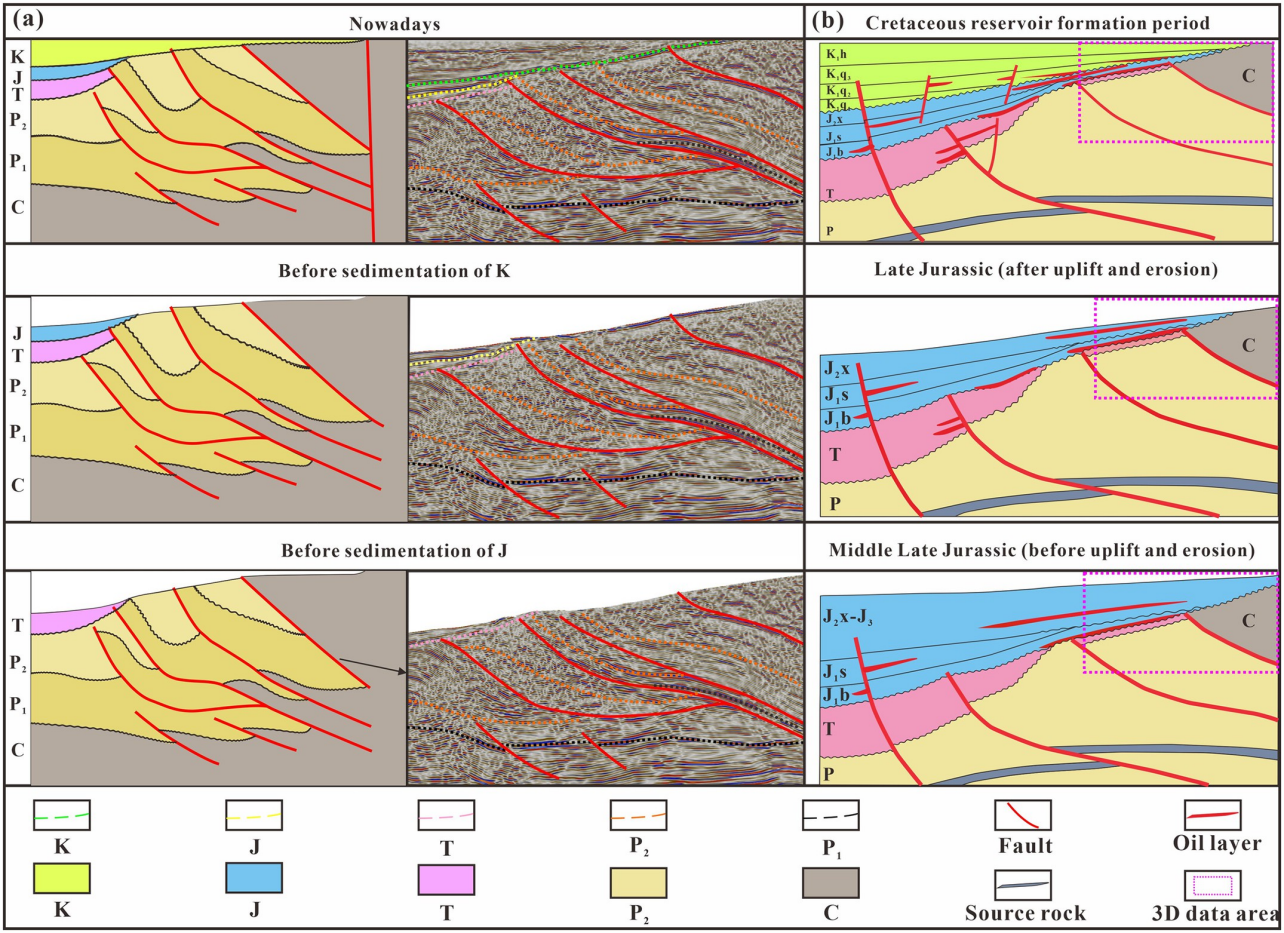
Tectonic evolution and reservoir formation evolution. (a) Structural evolution in the study area, (b) the impact of structural evolution on oil and gas migration in the study area.

The Hala’alate Mountain area has experienced a complex evolution process and formed a complex fault system. The shallow fault system in the study area controls the vertical migration of hydrocarbons (Fig 15a). Late Jurassic tectonic activity caused the oil source faults to connect the sand bodies of the Badaowan Formation and the Xishanyao Formation. The late Cretaceous oil source fault was reactivated, and the fault extended upward to the sand bodies of the Qingshuihe Formation in the Lower Cretaceous. Moreover, the unconformity between the upper and bottom conglomerates in the Hala’alate Mountain area exhibits notable heterogeneity, notable cementation, poor physical properties, no horizontal transport ability, and no long-distance hydrocarbon migration ability (Fig 15b). The first and second members of the Xishanyao Formation (JSQ1-2) are lowstand domains. The fan delta front channel sand bodies at the edge of the lake basin provide good reservoir conditions, and the mudstone in the lake transgressive domain at the top could be used as a cap rock. The third member of the Xishanyao (JSQ3) and Qingshuihe Formations (KSQI) are both lake transgressive domains. Only the fan delta front sand body on the basin edge and the shore shallow lake beach bar sand body can be used as reservoirs. The mudstone of the shore shallow lake facies and the weathered mudstone of the unconformity interface can be considered good caprocks.

**Fig 16:**
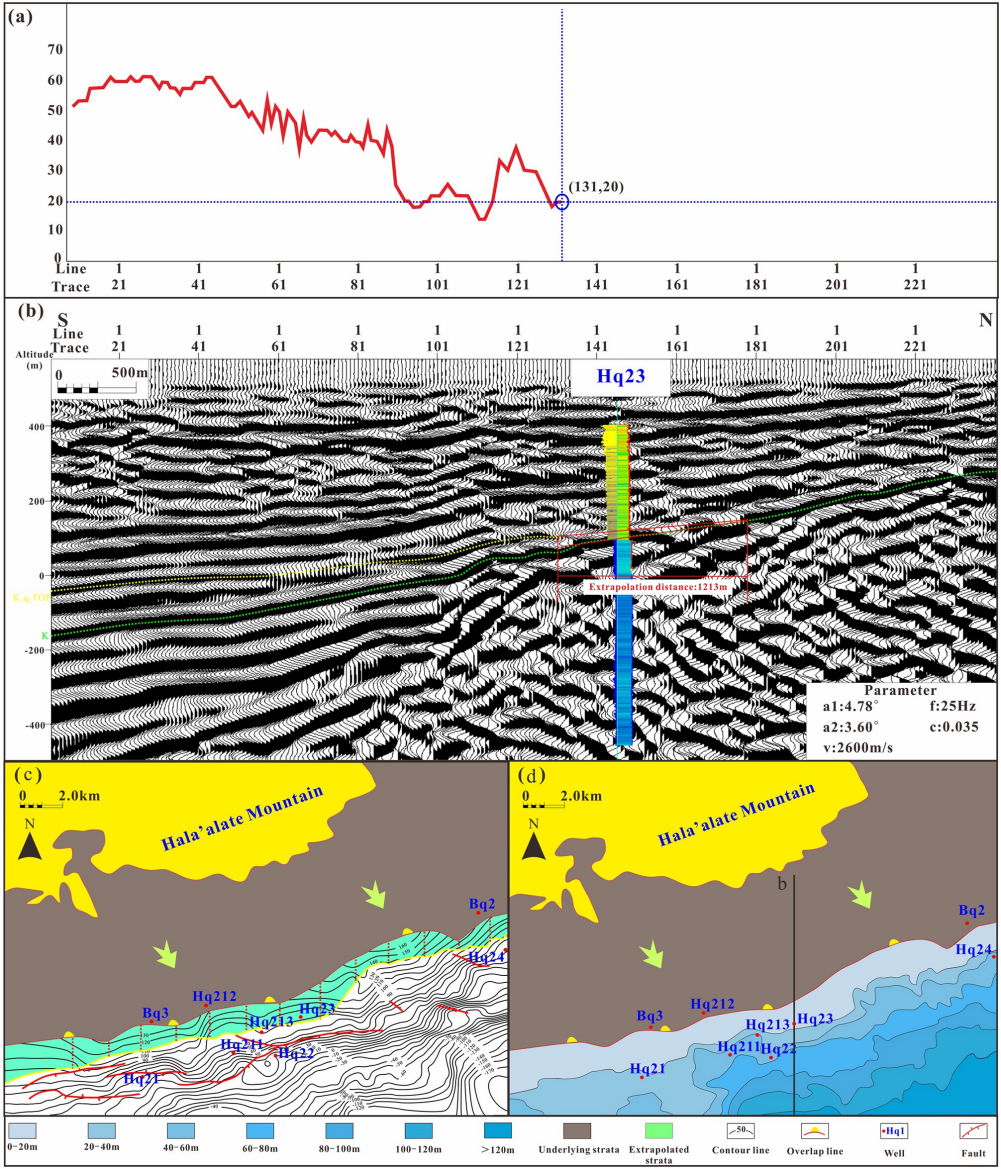
Stratigraphic distribution after angle extrapolation. (a) Single-channel half energy attribute. (b) Prediction results of the formation pinch-out line passing through well Hq23. (c) Structural map of the top surface of the second section of the Qingshuihe Formation. (d) Stratum thickness map of the second section of the Qingshuihe Formation.

**Fig 17:**
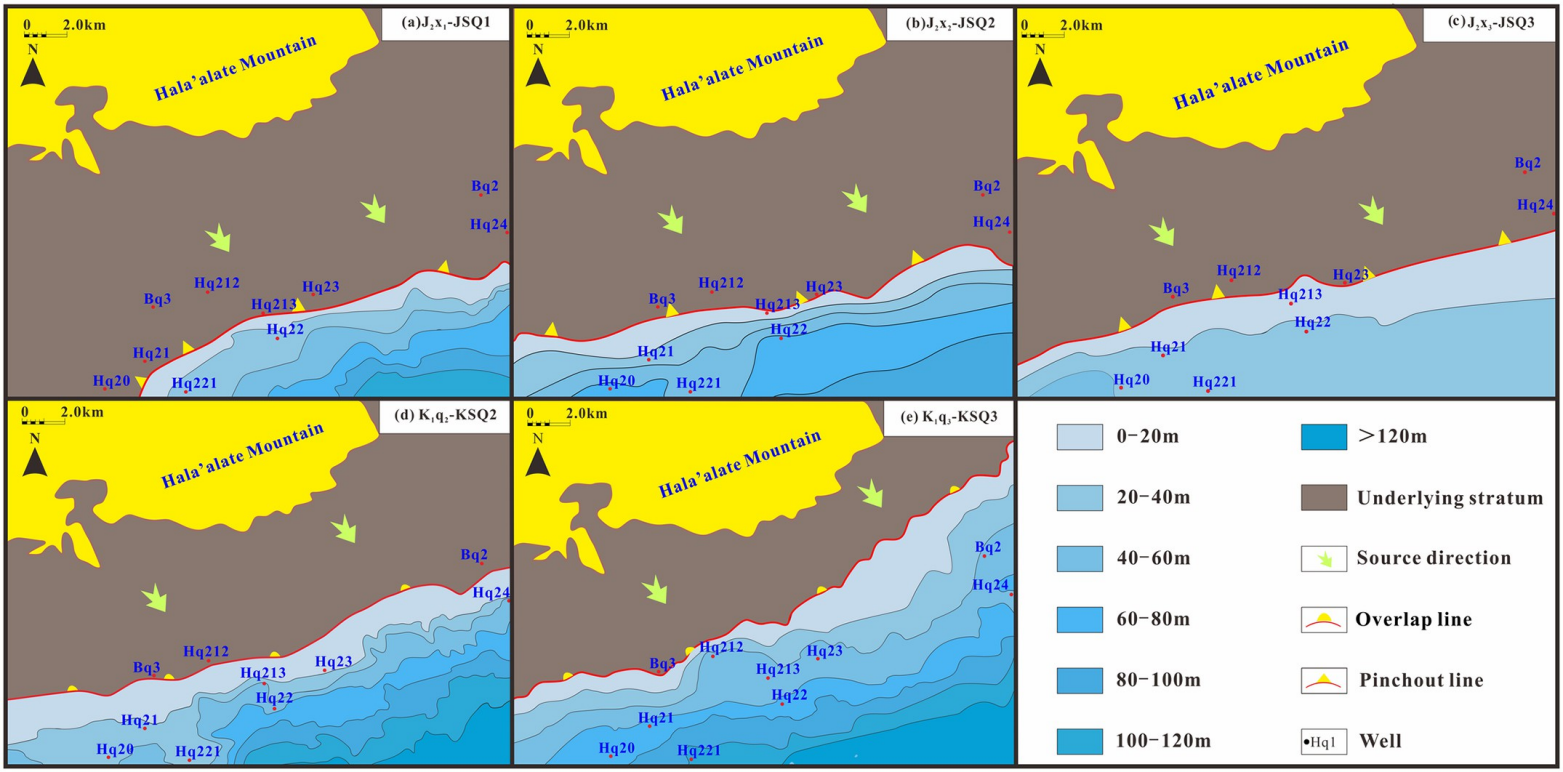
Evolutionary map of the Cretaceous residual strata on the southern margin of Hala’alate Mountain. (a) and (b) JSQ1-2 (LST), affected by the Yanshan Movement, suffered erosion due to strong uplift source supply and thick sedimentation. (c) JSQ3 (TST), developed short-term lake intrusion and small-scale fan delta sedimentation. (d) and (e) KSQ2-3 (TST) developed continuous lake intrusion and developed fan delta sedimentation, expanding the sedimentary area from bottom to top.

The general dip angle of the unconformity surface in the study area is approximately 5° and the extrapolation distance is approximately 1000 m. Using the seismic profile of well Hq23 the single-channel half energy attribute curve of the Qingshuihe Formation is identified, and the extrapolation distance between the seismic reflection cusp and the actual cusp is calculated to be 1229 m (Fig 16a and 16b). The thickness of the Hq23 Qingshuihe Formation is 17 m, difficult to identify via seismic data. Well Hq213 is close to the formation pinch-out line and the remaining thickness is 46 m. By extrapolation, the formation boundary is more realistic and reliable (Fig 16c and 16d). After extrapolation, the sedimentary boundary of the fan delta front moved northward. The strata are thick in the southeast and thin in the northwest. The well blocks Hq21 and Hq22 of the Xishanyao Formation are controlled by the cutting line to the northwest. The Hq23 and Hq21 well blocks of Cretaceous strata overlap to the northwest. The reservoir is controlled by the sedimentary sand body at the formation boundary. Hydrocarbons are preferentially injected into thick sand bodies with good physical properties in the channel of nose uplift structures. The distribution map of residual strata in the study area has been obtained through angle extrapolation. In the JSQI area, the sedimentary range has reduced due to uplift caused by the Yanshan movement. On the other hand, in the KSQI area, sedimentation is primarily influenced by tectonic subsidence and sediment supply, resulting in an expanded sedimentary range (Fig 17). In summary, the distribution characteristics of traps in the study area are controlled by the distribution of sedimentary sand bodies, stratigraphic super erosion lines and structures.

### Main trap types

**Fig 18:**
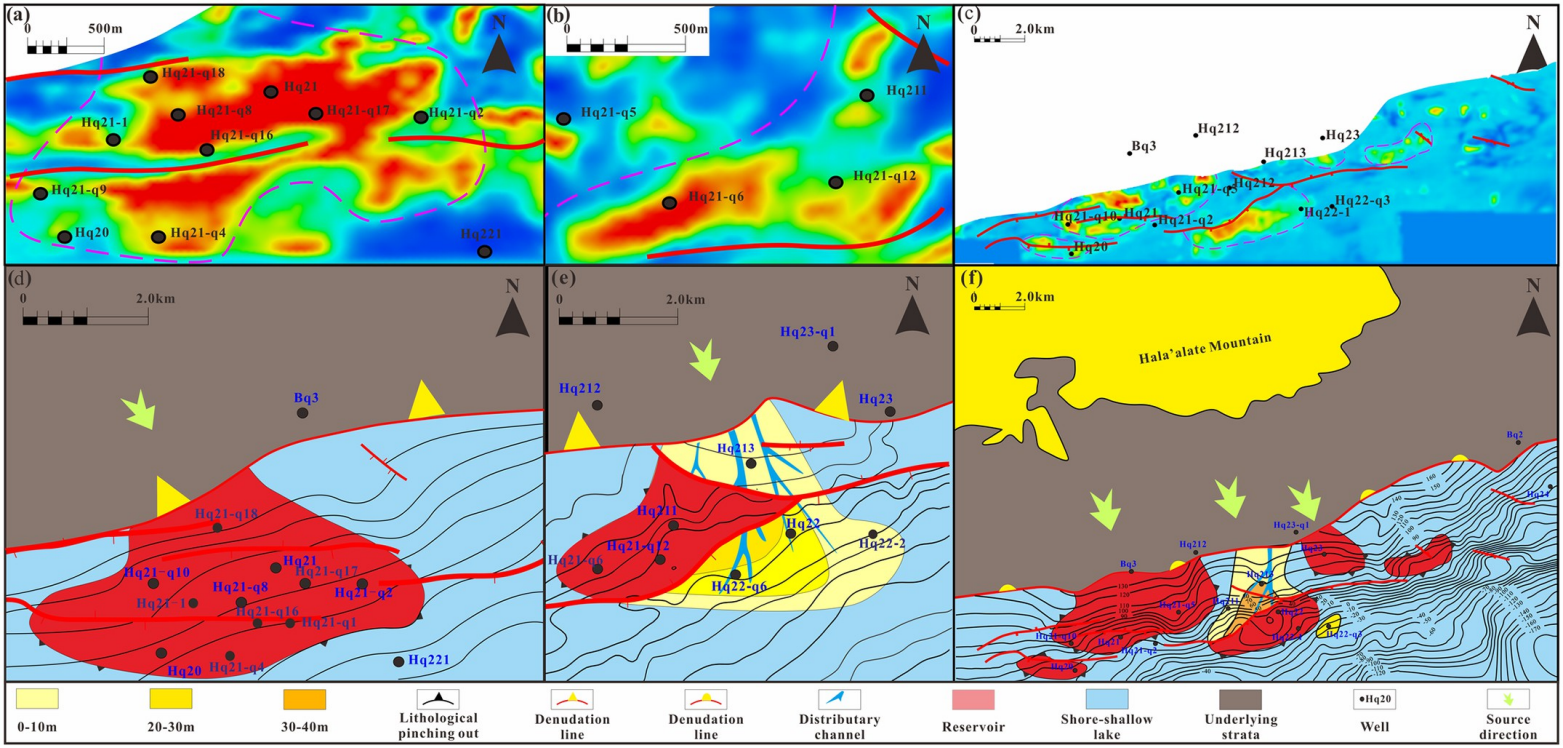
Reservoir plan and Poisson’s ratio inversion response in the study area. (a). Poisson’s ratio inversion stratigraphic slice of the third member of the Xishanyao Formation (JSQ3) in the Hq21 well area, (b). Poisson’s ratio inversion stratigraphic slice of the third member of the Xishanyao Formation (JSQ3) in the Hq22 well area, (c) Poisson’s ratio inversion stratigraphic slice of the second member of the Qingshuihe Formation (KSQ2), (d). Reservoir plan of the third member of the Xishanyao Formation (JSQ3) in the Hq21 well area, (e). Reservoir plan of the third member of the Xishanyao Formation (JSQ3) in the Hq22 well area, (f) Reservoir distribution prediction of the second member of the Qingshuihe Formation (KSQ3).

**Fig 19:**
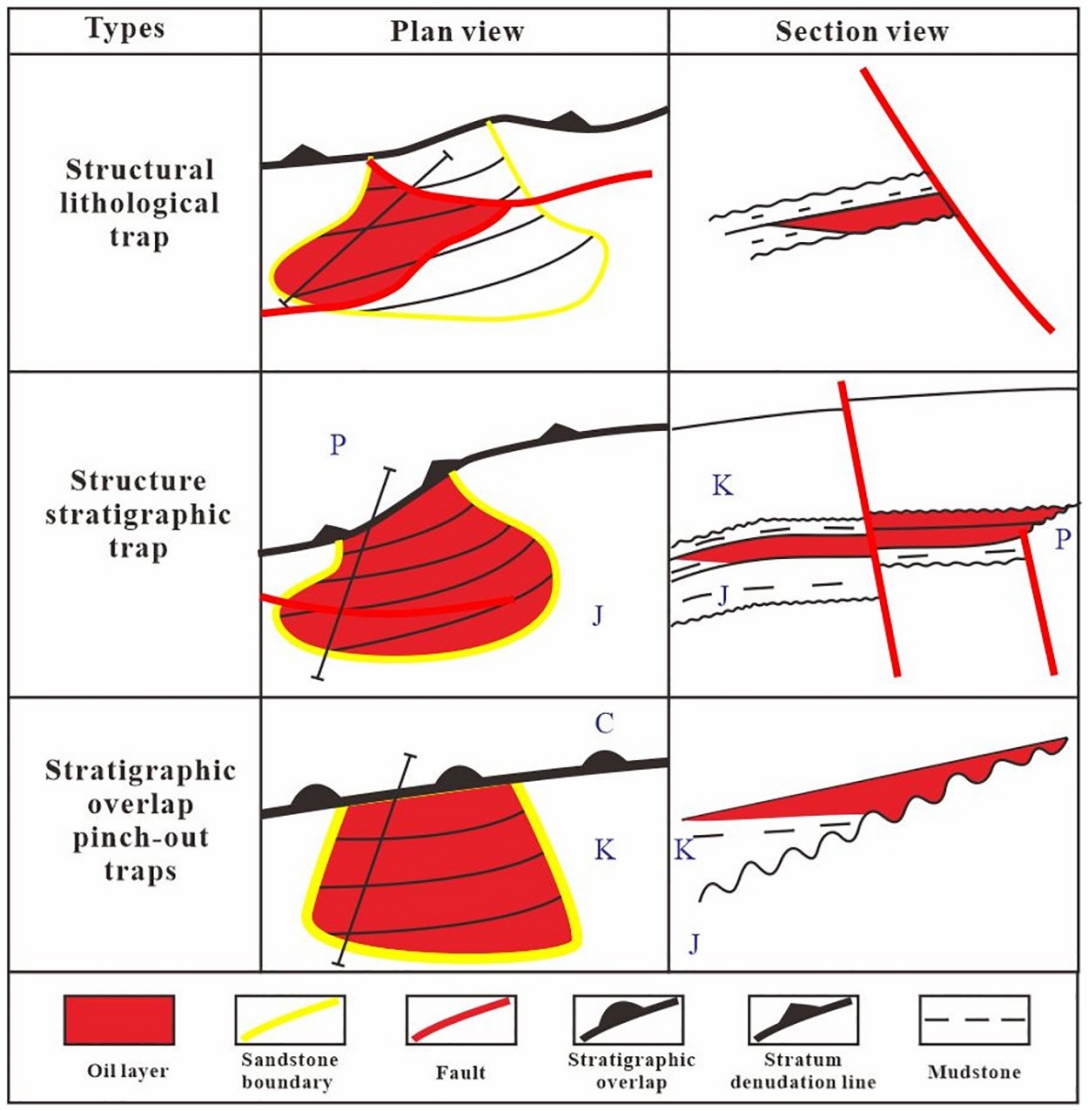
Diagram of the reservoir formation pattern in the study area.

Poisson’s ratio has strong representativeness for lithological and hydrocarbon prediction [55]. The third member of the Xishanyao Formation (JSQ3) and the second member of the Qingshuihe Formation (KSQ2) are favorable areas in the study area. Confirmed hydrocarbon responses were observed in the cores of the third member of the Xishanyao Formation in wells Hq21 and Hq21-q6, both located in the yellow-red low Poisson’s ratio area. The northern part of the Hq21 well block is defined by the Hq21 North fault, while the northwestern region is restricted by the sedimentary sand body boundary and the stratigraphic boundary, forming a structural stratigraphic reservoir. In the southern section of the Hq21 well block, especially Hq21-q4, structural lines and faults play a controlling role, forming structural lithologic reservoirs (Fig 18a and 18b). Hq211, Hq21-q12 and Hq21-q10 show a yellow-red response on Poisson’s ratio slices (Fig 18c and 18d). The southern and northern parts of the third member of the Xishanyao Formation (JSQ3) are bounded by faults and the western member is bounded by sand body boundaries forming a structural lithologic reservoir. Fan delta front sand bodies are widely developed near the pinch-out line of the second member of the Qingshuihe Formation (KSQ2). Controlled by lithology and the pinch-out line, the Hq21 and Hq23 well blocks are presumed to be lithologic stratigraphic trap reservoirs (Fig 18e and 18f). In summary, the study area is mainly composed of structural stratigraphic traps, structural lithologic traps, and stratigraphic overlap pinch-out traps (Fig 19).

## Discussion

### Factors influencing the identification of the super denudation line

**Fig 20:**
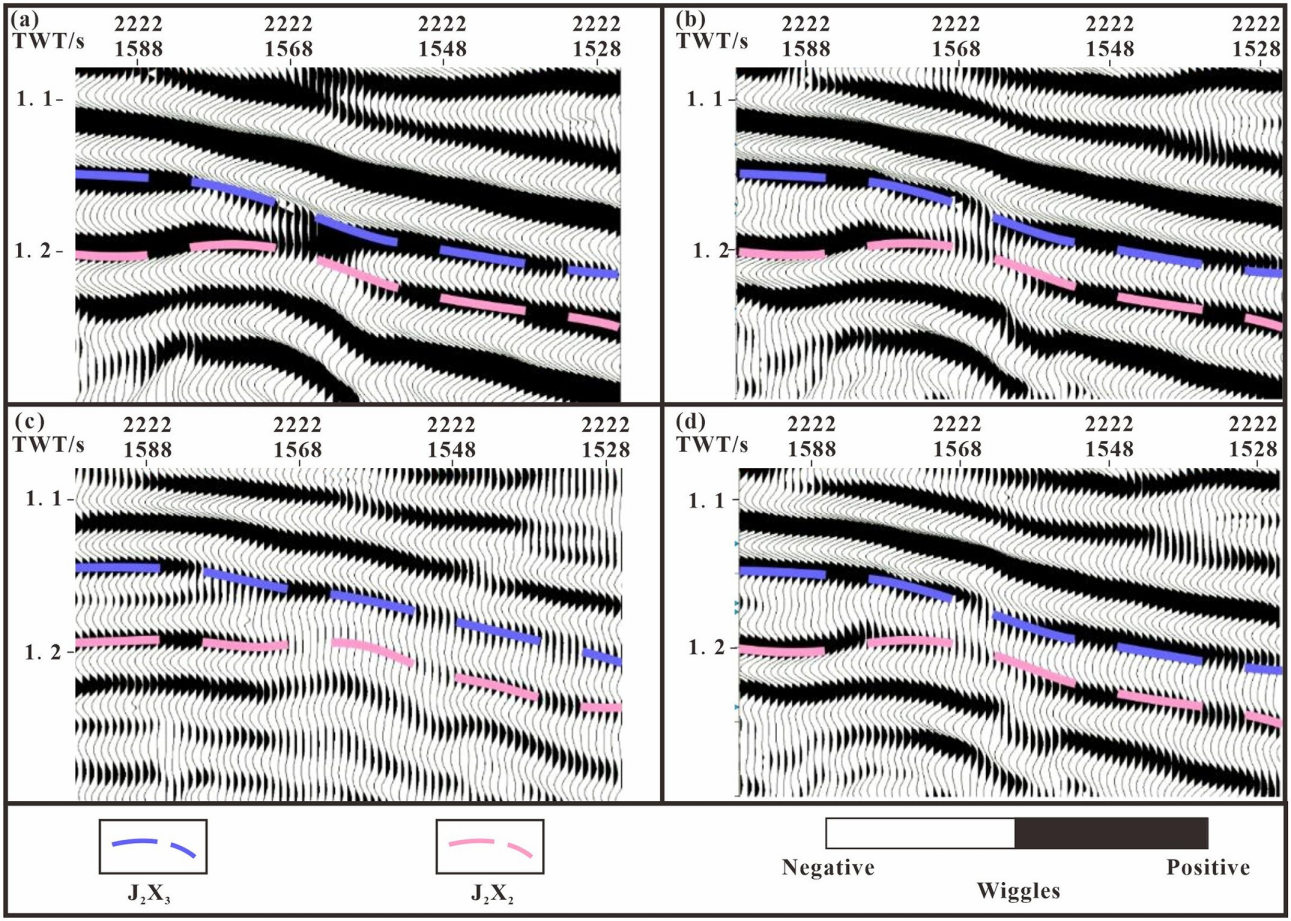
Comparison of spectrum decomposition effect. (a) 25Hz, (b) 35Hz, (c) 45Hz, (d)55Hz.

The propagation of seismic waves in rock formations is affected by the nature, composition, density, burial depth, and other factors of the rock formations [56, 57]. When tracking horizons, we first consider the original data volume. We hope that the tracked seismic event events are smooth, the reflection amplitudes are similar, and the waveforms are similar. However, there are still problems of large amplitude fluctuations and high noise in the tracking process. Therefore, horizon tracking is assisted by time-frequency analysis technology. The boundary frequency method and the adjacent frequency method are used to determine and select the optimal frequency. Compared with the data volumes of 25 Hz, 35 Hz and 55 Hz, 45 Hz has better continuity and stability, highlights the amplitude intensity of the target main frequency range, and simultaneously suppresses the amplitude response of other frequency (Fig 20). However, for pinch-out formations with low angles or weak reflected energy, identifying pinch-out points according to changes in the event axis is particularly difficult [58]. Therefore, we first determined the initial position of the pinch-out line before performing angle extrapolation according to the seismic response characteristics; however, this position was still affected by factors such as the formation dip angle and seismic data quality. For example, Hq23 was preliminarily identified as being outside the pinch-out line but was proven to be in the second member of the Qingshuihe Formation according to logging core and other data. To further study the relationship between the error and pinch-out angle, the forward model and extrapolation results were used for analysis (Table 3).

**Table 3:** K_1_q_2_ extrapolation distance calculation.

Predicted thickness (m)	Correction coefficient	Line location	Dominant frequency (Hz)	a_1_(°)	a_2_(°)	V(m/s)	Extrapolation distance(m)
26.93	-0.010	1945	24	3.1	2.2	2610.7	1744.216
22.81	-0.060	1995	27	5.5	3.5	2666.1	747.8403
31.05	-0.001	2045	22	4.5	3.2	2734.5	1364.078
26.93	0.045	2095	27	4.2	2.4	2783	782.1952
26.93	0.029	2145	26	4.1	2.9	2723	1210.866
22.81	-0.020	2195	28	4.8	3.8	2606.7	1353.099
22.81	-0.024	2245	27	5.4	4	2524.2	973.5703
26.93	0.007	2295	24	5.2	4.3	2567	1678.632
22.81	-0.056	2345	27	4.2	3	2610.7	1218.419
31.05	0.094	2395	23	5.9	4.2	2610.7	867.1735
31.05	-0.001	2445	21	4.8	3.3	2610.7	1182.21
26.93	-0.010	2495	24	5.6	2.3	2610.7	474.4127
31.05	0.094	2545	23	3.3	2	2610.7	1140.445

where a_1_- dip angle of the unconformity surface, °; a_2_- overburden dip angle, °; V—formation velocity, m/s.

**Fig 21:**
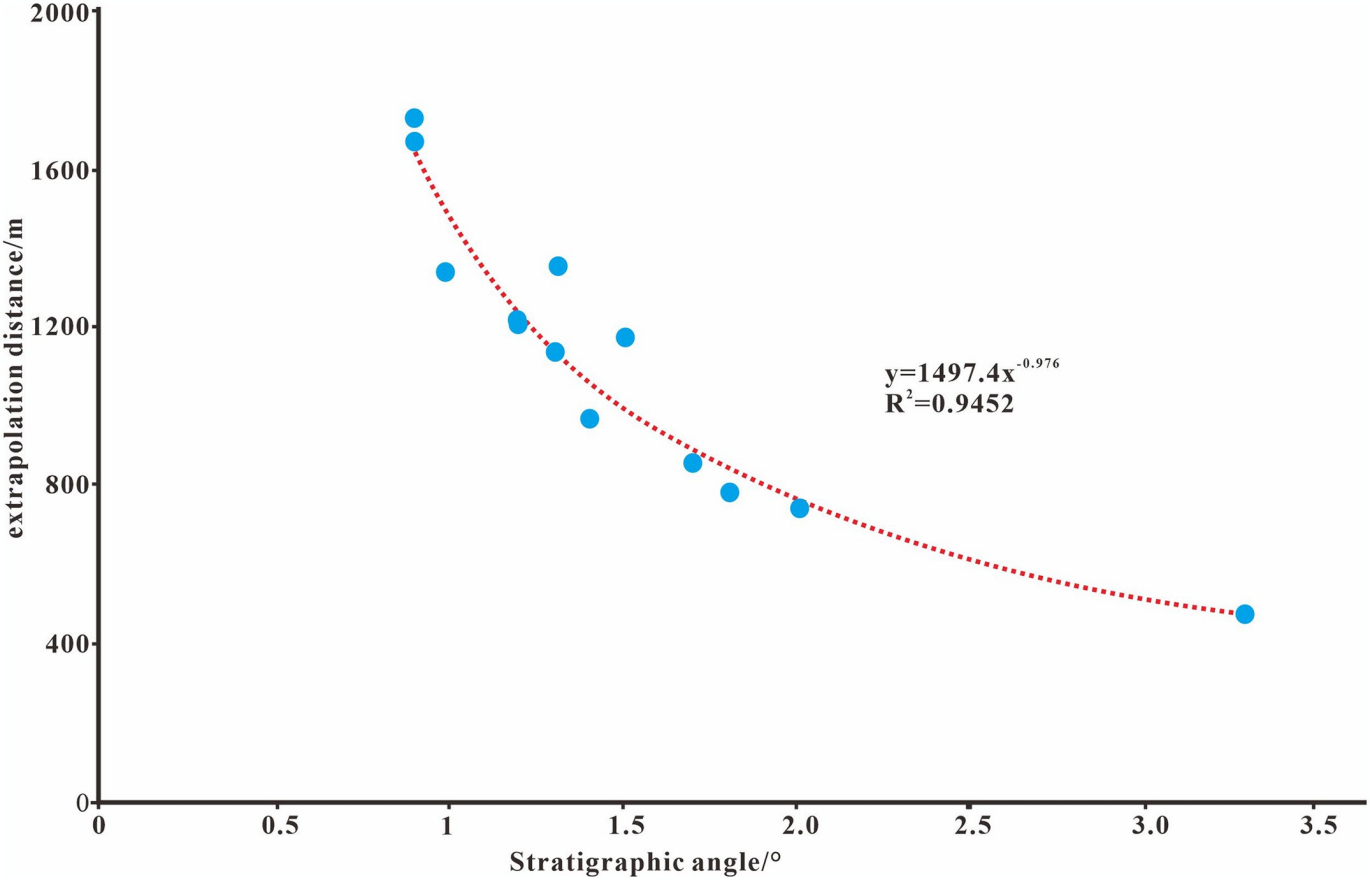
Error curves for the stratum angle and stratum overlap pinch-out point.

As determined using the error statistics, the position of the stratum overlap pinch point changes with the dip angle of the unconformity surface and the stratum dip angle. The greater the dip angle difference between the stratum and the unconformity surface the smaller the error between the pinch point and seismic reflection and vice versa. When the dip angle difference is 8° the error between the cusp and seismic reflections is 153.3 m, but when the dip angle difference is 23° the error is only 45 m. There is an obvious power exponential linear relationship between the difference in dip angle and the identification error. Using the actual data from the study area, the relationship between the pinch-out point errory and the pinch-out angle X in the study area is y = 1497.4x^-0.976^ (Fig 21).

### Validation of Poisson’s ratio genetic inversion for stratigraphic trap prediction

**Fig 22:**
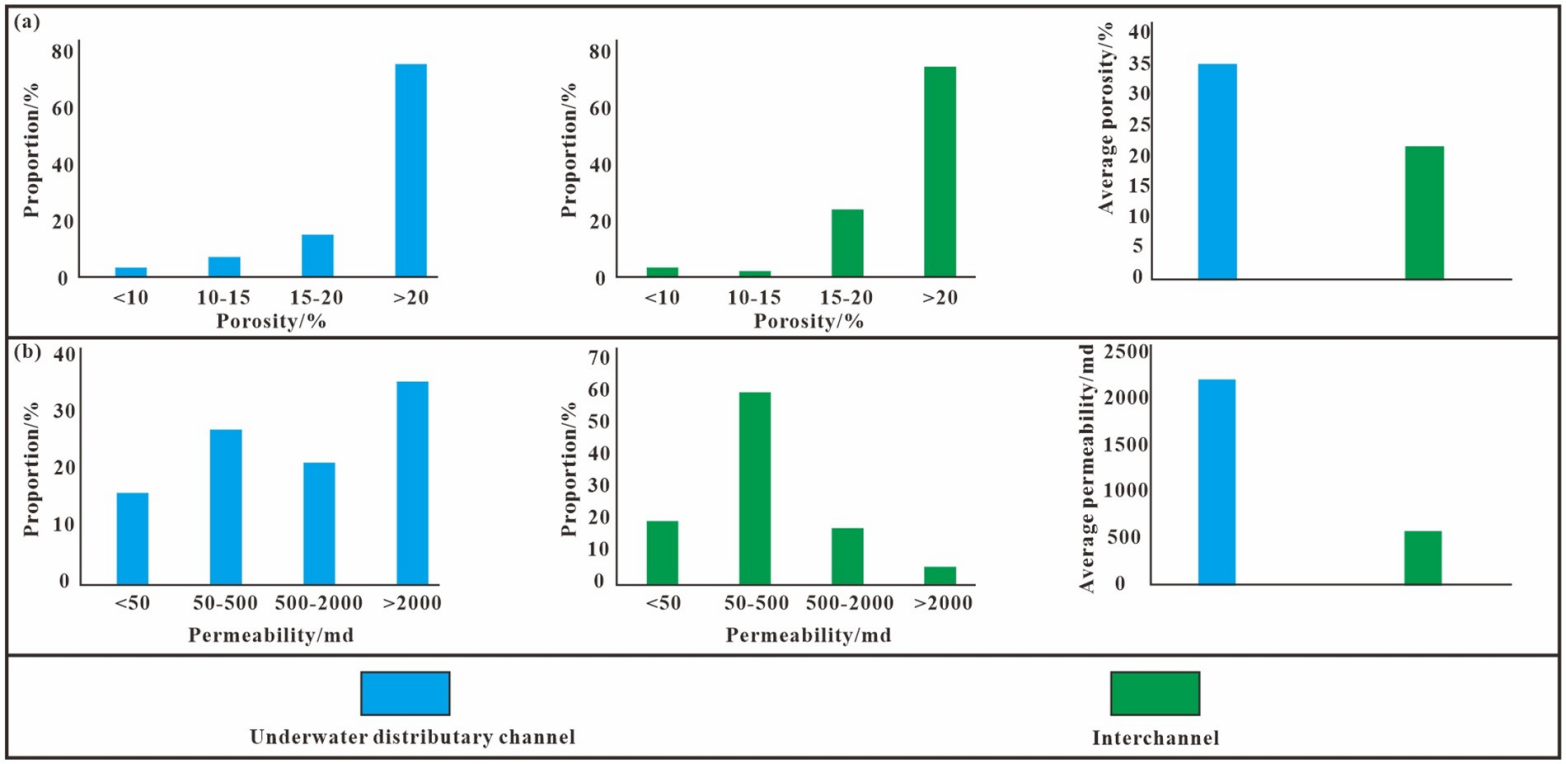
Distribution map of average reservoir physical property values of different sedimentary facies in the Jurassic cretaceous system of the study area. (a) Porosity of different sedimentary facies types, (b) permeability of different sedimentary facies.

**Fig 23:**
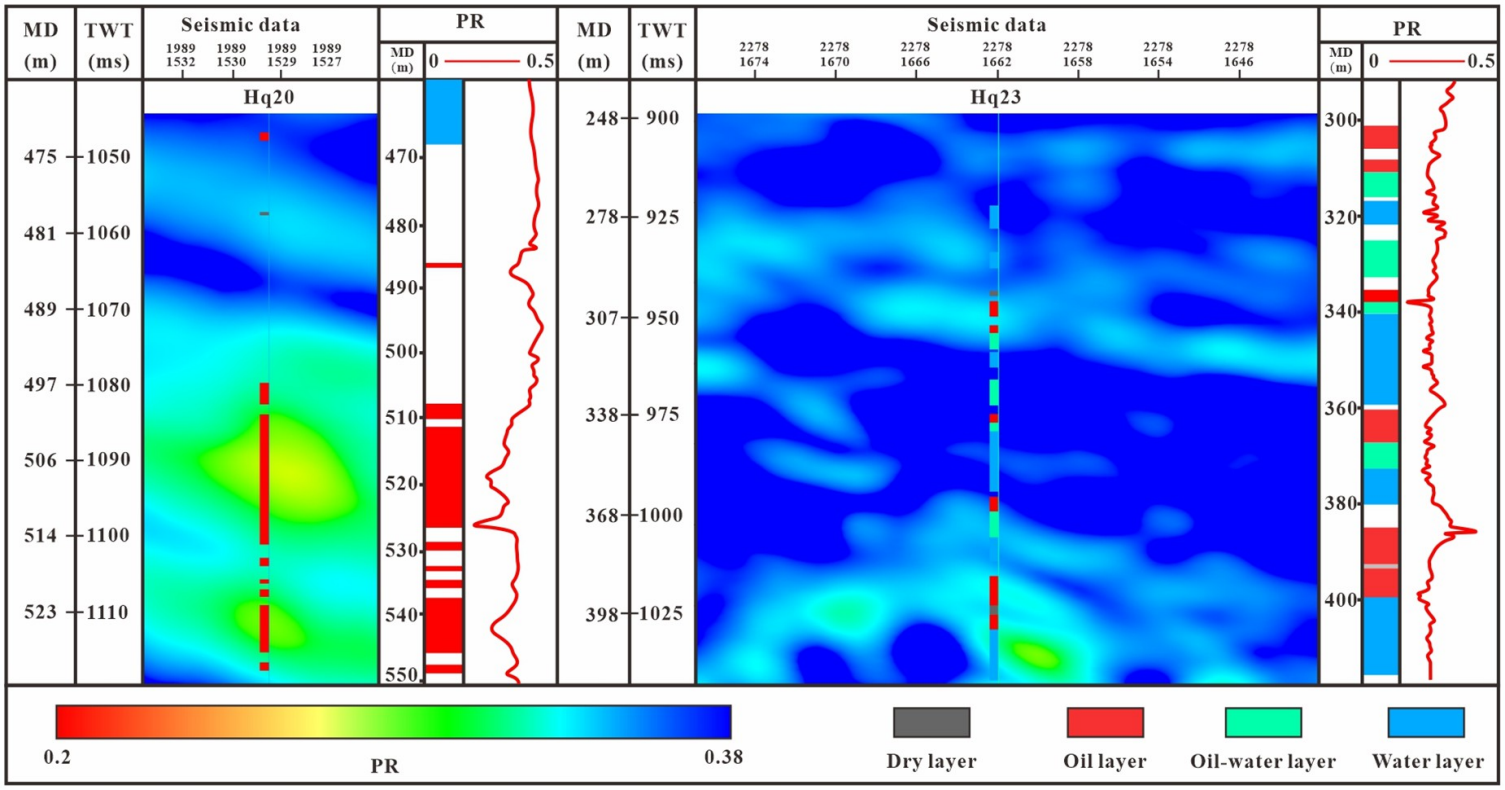
Comparison of Poisson’s ratio genetic inversion results.

In this study, Poisson’s ratio genetic inversion was performed on poststack seismic data to detect hydrocarbons in the target formation. Sedimentary facies mainly control the quality of reservoirs [59]. The main sedimentary microfacies in the study area are underwater distributary channels and interchannel. The underwater distributary channel reservoir is characterized by high porosity and permeability, with an average porosity of 35% and an average permeability of 2143x10^-3^ μm^2^. Reservoirs between underwater distributary channels usually have high porosity but low permeability (Fig 22). The plane distribution of these two microfacies is consistent with the Poisson’s ratio inversion slice. To verify the reliability of the inversion results, Hq20 and Hq23 were selected as postverification wells. The interwell inversion results of the interval are consistent with the logging and production conditions (Fig 23). The average thickness of each oil layer in well Hq23 in the Qingshuihe Formation is 4.9 m. The cumulative thickness of the reservoir in the Qingshuihe Formation in Hq20 reaches 34.6 m, and the Poisson’s ratio response is good. The reservoir in well Hq23 is thin, and a water layer has developed. A yellow-green warm response can still be identified south of Hq23 and is speculated to be a stratigraphic overlap controlled by the stratigraphic boundary. The outer expansion of the formation boundary improves the prediction accuracy of formation overlap reservoirs, provides a new idea for boundary-type reservoirs, and has a wide range of applicability for the same type of reservoir.

## Conclusion

This study, through the revision of the angle extrapolation formula and the use of Poisson ratio genetic inversion, has reduced errors in seismic data caused by resolution issues. However, the study did not directly enhance the resolution of the seismic data. Due to the prohibitively high cost of reacquiring seismic data, there are still many avenues to explore in the realm of stratigraphic pinch-out oil reservoirs. The presence of strong reflections at unconformity interfaces creates masking effects on pinch-out reflections. Future research can focus on extracting background information from seismic data, mitigating masking effects, in order to accurately obtain stratigraphic pinch-out information.

A quantitative description of the formation pinch-out line is realized. The inconsistency of the pinch-out point of the seismic reflection event axis compared to the overburden boundary of the actual formation is reconciled by comprehensive use of seismic attribute identification and an improved included angle extrapolation method. The closer to the pinch-out line, the smaller the formation thickness, and the trap is difficult to identify. Poisson’s ratio genetic inversion is used to characterize the spatial distribution characteristics of stratigraphic reservoirs.

Stratigraphic reservoirs are controlled by factors such as stratigraphic overbreaklines, sedimentary facies belts, sequence stratigraphy evolutionand structures. The traps in the south edge of Hala’alate Mountain are mainly developed in fourth-order sequences, and the erosional unconformity traps in the third member of the Xishanyao Formation (JSQ3) and the overlap traps unrelated to the erosional unconformity in the second member of the Qingshuihe Formation (KSQ2) are identified.

The stratigraphic reservoir in the south edge of Hala’alate Mountain includes the stratigraphic lithologic reservoirs in the Hq21 well area of the third member of the Xishanyao Formation (JSQ3) and the stratigraphic overlap reservoir in the second member of the Qingshuihe Formation (KSQ2). Poisson’s ratio genetic inversion reveal that the channel sand bodies and micronasal uplift structures in front of the fan delta in the study area provide good reservoir space for hydrocarbon accumulation.

## Supporting information

S1 Fig Particle composition of detrital rocks (in Fig 6). (XLSX)

S2 Fig Drilling thickness and half energy (in Fig 12). (XLSX)

S3 Fig Poisson’s ratio and GR (in Fig 13). (XLSX)

S4 Fig Porosity and permeability (in Fig 22). (XLSX)

S5 Fig Stratum thickness data (in Figs 16 and 17). (XLSX)

S1 Table Statistical data (in Table 3). (XLS)
